# Age-group differences in diffusion MRI characteristics of cortico-cerebellar tracts and their association with bimanual performance

**DOI:** 10.3389/fnagi.2026.1933331

**Published:** 2026-09-17

**Authors:** Maud Beeckmans, Shanti Van Malderen, Sara Magalhães Ferreira, Melina Hehl, Louise Emsell, Stefan Sunaert, Ahmed Radwan, Koen Cuypers

**Affiliations:** 1 Neuroplasticity and Movement Control Research Group, Rehabilitation Research Institute (REVAL), Hasselt University, Diepenbeek, Belgium; 2 Department of Imaging and Pathology, Translational MRI, KU Leuven, Leuven, Belgium; 3 KU Leuven, Brain Institute (LBI), Leuven, Belgium; 4 Department of Rehabilitation Sciences, KU Leuven, Leuven, Belgium; 5 Geriatric Psychiatry, University Psychiatric Center KU Leuven, Leuven, Belgium; 6 Department of Neurosciences, KU Leuven, Neuropsychiatry, Research Group Psychiatry, Leuven, Belgium; 7 Movement Control and Neuroplasticity Research Group, Department of Movement Sciences, Group Biomedical Sciences, KU Leuven, Leuven, Belgium

**Keywords:** aging, cerebellum, cortico-cerebellar loop, diffusion MRI, fixel-based analyses, motor control

## Abstract

**Introduction:**

Aging is associated with alterations in white matter structure and decline in bimanual performance. These parallel changes may be interrelated, as previous studies have linked cerebral white matter properties to both motor and cognitive function. The cerebellum plays a central role in complex motor coordination and higher-order processes, yet age-related differences in cortico-cerebellar pathways remain insufficiently understood. This study therefore examined two major cortico-cerebellar tracts — the dentato-rubro-thalamo-cortical tract (DRTT) and the cortico-ponto-cerebellar tract (CPCT) — to assess age-related differences in white matter properties and their association with bimanual motor performance.

**Methods:**

Diffusion Magnetic Resonance Imaging data from 46 younger and 46 older healthy adults were analyzed using a state-of-the-art fixel-based analysis approach to derive fiber-specific metrics reflecting micro- and macrostructural axonal properties.

**Results:**

An overall reduction in fiber density and cross-section was observed in older adults, primarily driven by macrostructural differences; alongside localized increases in these structural properties in specific regions. No significant associations were found between cortico-cerebellar white matter properties and bimanual motor performance.

**Discussion:**

These findings corroborate the general patterns of age-related white matter decline while underscoring the importance of within-tract analyses. Although cortico-cerebellar pathways demonstrate age-related structural differences, their contribution to age-related alterations in bimanual coordination performance in healthy aging may be limited or obscured by tract-level averaging masking regional effects and the multifactorial nature of bimanual motor coordination, which reflects the interaction of distributed neural and peripheral systems. The observed regional increases of fiber-specific measures in older adults may reflect age-related adaptations. Yet, their functional significance remains uncertain and warrants further investigation.

## 1 Introduction

As global life expectancy rises (World Health Organization, 2025),^1^ age-related decline in motor control poses a growing challenge for maintaining functional independence, and, ultimately, quality of life (e.g., Seidler et al., 2010). In particular, complex motor control that relies on precise temporal coordination between both hands deteriorates in older people, compared to younger individuals (e.g., Seidler et al., 2010; Maes et al., 2017; Boisgontier et al., 2018). While peripheral factors such as age-related musculoskeletal changes (e.g., Boros and Freemont, 2017; Frontera, 2017) contribute to this deterioration in motor control, converging evidence suggests that age-related alterations in the brain and broader central nervous system play a critical role (e.g., Seidler et al., 2010; Clark and Taylor, 2011; Inoue and Nishimune, 2023).

In particular, age-related changes in brain structure and function have been widely documented (Seidler et al., 2010; Maes et al., 2017). For instance, neuroimaging studies reveal reductions in gray and white matter (GM and WM, respectively) volume (Fjell and Walhovd, 2010; Walhovd et al., 2011; Blinkouskaya and Weickenmeier, 2021), altered task-related activation patterns (e.g., Cabeza, 2002; Naccarato et al., 2006; Heuninckx et al., 2008; Seidler et al., 2010; Monteiro et al., 2017), and diminished structural and functional connectivity within, among others, sensorimotor and associative networks in older compared to younger adults (Andrews-Hanna et al., 2007; Seidler et al., 2010; Goh, 2011; Zhao et al., 2015; Damoiseaux, 2017).

Notably, age-related decline in WM (i.e., the part of the central nervous system that contains axons connecting different brain regions) properties, particularly in tracts supporting motor control, have been linked to poorer motor performance (e.g., Koo et al., 2012; Bruijn et al., 2014; Serbruyns et al., 2015; Serbruyns et al., 2016; Oschwald et al., 2021) and motor learning (e.g., Schulz et al., 2014) in older adults.

However, studies investigating age-related differences in WM properties have predominantly focused on cerebral WM, revealing widespread age-related differences in WM properties and network organization and their associations with motor and cognitive performance deficits (Madden et al., 2009; Serbruyns et al., 2015; Damoiseaux, 2017; Zivari Adab et al., 2020; Rasooli et al., 2021; Rasooli et al., 2023). In contrast, far less attention has been paid to age-related differences in the structural connections between the cerebellum and cortical motor-related brain regions (e.g., Bernard, 2022), despite their established importance for motor coordination and learning (Doyon et al., 2003; Schmahmann et al., 2004; Ramnani, 2006; Ito, 2008; Balsters et al., 2010; Ramnani, 2012; Apps and Watson, 2013; Koziol et al., 2014; Schulz et al., 2014).

Beyond the cerebral cortex, the cerebellum is increasingly recognized as a critical contributor to age-related changes in motor and cognitive functioning (e.g., Arleo et al., 2024). Previous studies have reported, for instance, age-related reductions in cerebellar GM and WM volume (e.g., Luft et al., 1999; Andersen et al., 2003; Raz et al., 2005; Bernard and Seidler, 2013), as well as altered functional and structural connectivity with cortical areas (Bernard et al., 2013; Liu et al., 2018; Hausman et al., 2020; Uwisengeyimana et al., 2020; Bernard et al., 2021; Patil et al., 2021; Hicks et al., 2024; Khalilian et al., 2024). Furthermore, recent analyses using graph-theory have shown that the cerebellum’s involvement in the brain’s “rich club” (i.e., a set of highly connected hub nodes) network declines with advancing age, alongside a reduction in the integration and segregation of cerebellar connectivity (Khalilian et al., 2024).

Despite these converging indications of age-related changes in the cerebellum, little is known about the impact of aging on the structural connections linking the cerebellum and cortical motor-related brain regions. The cerebellum and cortical brain regions are connected by two major reciprocal pathways (i.e., cerebello-cortical loops): the dentato-rubro-thalamo-cortical tract (DRTT) and the cortico-ponto-cerebellar tract (CPCT). The DRTT conveys efferent signals, originating in the cerebellar hemisphere, from the dentate nucleus to contralateral motor areas and prefrontal cortex via the superior cerebellar peduncle, red nucleus and thalamus (e.g., Hoover and Strick, 1999; Middleton and Strick, 2001; Kelly and Strick, 2003). Conversely, the CPCT, transmits afferent input from several cortical regions (including premotor, prefrontal and posterior parietal areas of the cerebral cortex) (ref. Bostan et al., 2013) to the cerebellum via the pons and middle cerebellar peduncle (e.g., Schmahmann et al., 2004; Ramnani, 2006; Balsters et al., 2010; Ramnani, 2012; Apps and Watson, 2013). Together, these reciprocal cerebello-cortical pathways support the integration of motor and cognitive information by continuously relaying efferent and afferent movement-related signals (including efference copies and sensory signals) between the cerebellum and cortex, thereby enabling error monitoring, predictive modeling, and the ongoing updating and execution of motor commands through the cortical motor regions (e.g., Ito, 1972; Wolpert et al., 1998; Miall et al., 2001; Miall and Reckess, 2002; Ito, 2006; Ebner and Pasalar, 2008; Tanaka et al., 2019). On this basis, both pathways play a central role in a wide range of non-motor and motor functions, including motor coordination and planning, movement regulation, and motor learning (Rispal-Padel et al., 1981; Koziol et al., 2014; Schulz et al., 2014; Dacre et al., 2021; Zhu et al., 2023; Ako et al., 2025). Accordingly, a disruption of these cortico-cerebellar connections has been associated with various neurological conditions, including ataxia, dystonia, and stroke, underscoring the importance of the structural properties of these cortico-cerebellar tracts for normal motor function (Schulz et al., 2015; e.g., Jang and Lee, 2021; Morigaki et al., 2021).

Diffusion-weighted magnetic resonance imaging (dMRI) offers a non-invasive method to approximate the structural properties of WM pathways *in vivo*. However, despite their functional importance, age-related changes in these two pathways and its implications for motor control remain largely unexplored. Specifically, while the DRTT and CPCT have been studied in a limited number of studies (e.g., Salmi et al., 2010; Palesi et al., 2015; Palesi et al., 2017), there are very few studies examining these tracts in the context of aging. In particular, Kraft et al. (2026) showed that aging was associated with increases in radial (RD) and axial diffusivity (AD) in cerebello-thalamo-cortical tracts, following a non-linear trajectory in which these increases become more pronounced with advancing age. Importantly, they only focused on fronto-cerebellar tracts using diffusion tensor imaging (DTI) and found that higher mean diffusivity, RD and AD were related to worse executive function in older, but not younger adults, suggesting that cerebellar–prefrontal connectivity contributes to age-related cognitive decline (Kraft et al., 2026). Along the same lines, reduced mobility in older adults was associated with reduced fractional anisotropy (FA) and AD in the superior and inferior cerebellar peduncles (Cavallari et al., 2013).

As compared to DTI, recent methodological advances in the field of diffusion-weighted MRI using multi-shell-multi-tissue constrained spherical deconvolution (MSMT-CSD) methods have substantially improved the characterization of complex WM tracts by enabling the reliable reconstruction of crossing fibers (Jeurissen et al., 2014; Dhollander et al., 2021), which are inadequately captured by conventional DTI-based measures. Using these approaches, previous studies have demonstrated the feasibility of reconstructing both DRTT and CPCT in healthy individuals (Palesi et al., 2017).

Given the established role of cortico-cerebellar pathways in bimanual coordination and adaptive motor control, age-related degeneration of the DRTT and CPCT may contribute to the decline in motor performance and slower motor learning commonly observed in older individuals. Therefore, the present study applied state-of-the-art dMRI and fixel-based analysis (FBA) to examine age-group differences in the structural properties of these cortico-cerebellar pathways and their relationship with bimanual motor performance.

In particular, our first aim (RQ1) was to identify age-group differences in the micro- and macrostructural WM of the DRTT and CPCT. Based on the broader WM aging literature, we hypothesize an overall decrease in these structural properties of the cortico-cerebellar tracts in older as compared to younger adults with aging, as seen in cortical tracts.

Our second aim (RQ2) was to evaluate whether age-related structural alterations of these cortico-cerebellar tracts were associated with differences in bimanual motor coordination. Here we hypothesize a positive relationship between the structural properties of the DRTT and CPCT and bimanual motor performance. Yet, the strength of this relation may differ in younger and older adults.

## 2 Materials and methods

### 2.1 Dataset description

This study combined data from two datasets collected by our research group (hereafter, Sub-study 1 and 2). Each sub-study included a group of younger (i.e., 20–40 years old) and a group of older adults (i.e., 60–80 years old). Sub-study 1 consisted of 50 participants of which four were excluded from the analyses due to brain anomalies (*N* = 2), insufficient data quality (*N* = 1) or missing data (*N* = 1). Sub-study 2 consisted of 54 participants of which eight were excluded due to brain anomalies (*N* = 2), insufficient data quality (*N* = 3) or missing data (*N* = 3). This resulted in 24 younger [11 males; median age = 23.50 and interquartile range (IQR) = 5.50] and 22 older adults [9 males; mean age = 66.86 and standard deviation (SD) = 4.84] included from Sub-study 1, and 22 younger (10 males; median = 29.00 and IQR = 8.75) and 24 older adults (12 males; median = 68.5 and IQR = 3.75) from Sub-study 2. An overview of these descriptives can be found in Table 1. Also, note that a subset of the data collected in both sub-studies have been used in previous studies (Van Malderen et al., 2025; Ferreira et al., 2026) to investigate different research questions based on data from different modalities [i.e., task-based functional magnetic resonance imaging (tb-fMRI) and functional magnetic resonance spectroscopy (fMRS), respectively].

**Table 1:** Summary metrics of the participants’ demographics.

SS	Age Gr.	Age (years)	Height (cm)	Weight (kg)	IPAQ (min)	MoCA	N (male/female)
1	Y	23.5 (5.5^) [20–39]	173.83 (9.96) [158–190]	68.04 (13.89) [48–97]	4410.75 (4105.5^) [360–20,478]	29.0 (2.0^) [26–30]	24 (11/13)
	O	66.86 (4.84) [60–75]	169.64 (10.44) [153–189]	66.5 (23.5^) [54–120]	4,716 (5990.25^) [1110–16,836]	28.5 (1.0^) [26–30]	22 (9/13)
Statistical comparison			*t* (44) = 1.40 *p* = 0.170	*W* = 232.00 *p* = 0.488	*W* = 222.00 *p* = 0.365	*W* = 297.00 *p* = 0.461	
2	Y	29.0 (8.75^) [25–40]	171.23 (6.54) [160–184]	69.53 (11.69) [45–94]	3346.73 (1994.6) [678–7218.5]	29.0 (2.0^) [26–30]	22 (10/12)
	O	68.5 (3.75^) [65–79]	169.17 (7.68) [155–180]	71 (14.23) [47–103]	5414.77 (2247.76) [2106–10765.5]	29.0 (1.25^) [26–30]	24 (12/12)
Statistical comparison			*t* (43) = 0.96 *p* = 0.341	*t* (44) = −0.38 *p* = 0.706	*t* (43) = −3.26 *p* = 0.002*	*W* = 268.00 *p* = 0.716	
1 + 2	Y	26.0 (7.75^) [20–40]	172.59 (8.51) [158–190]	68.75 (12.77) [45–97]	3,726 (4062^) [360–20,478]	29.0 (2.0^) [26–30]	46 (21/25)
	O	68.13 (4.58) [60–79]	169.4 (9.03) [153–189]	68 (19.75^) [47–120]	4,797 (4002^) [1110–16,836]	29.0 (1.0^) [26–30]	46 (21/25)
Statistical comparison			*t* (89) = 1.73 *p* = 0.860	*W* = 976.00 *p* = 0.524	*W* = 715.0 *p* = 0.011*	*W* = 1132.5 *p* = 0.427	

Mean and standard deviations (SD) or median and interquartile range (IQR) are reported for normally and non-normally distributed group variables, respectively, in the format ‘mean (sd) [min—max]’ or ‘median (IQR^) [min—max]’. The statistical comparison column contains the results from the statistical tests comparing younger and older adults. Unpaired t-tests and Wilcoxon Rank Sum tests are reported for normally distributed data and non-normally distributed data, respectively, where: *t*(degrees of freedom) = T-statistic, *W* = Wilcoxon statistic, and *p* = *p*-value associated with the respective test. Significance is defined at a level of 0.05 and indicated by ‘*’ in bold. Age Gr., Age group; cm, centimeter; IPAQ, International Physical Activity Questionnaire; kg, kilogram; min, minutes of moderate or intense physical activity per week as measured by the IPAQ; MoCA, Montreal Cognitive Assessment; N, number of participants; O, older adults; SS, sub-study; Y, younger adults.

The data for the two sub-studies were collected on the same magnetic resonance imaging (MRI) scanner with the same acquisition protocol for the dMRI data (see MRI acquisition parameters below for more details). Furthermore, participants in both sub-studies completed the bimanual tracking task (BTT) (Sisti et al., 2011) as a measure of motor performance. Importantly, the BTT familiarization phase and task conditions differed slightly across sub-studies (see section “Bimanual Tracking Task” for more detailed information).

Given the uniformity in dMRI data, the two sub-studies were pooled to evaluate the age-group differences in the structural properties of the cortico-cerebellar tracts (RQ1). However, given the difference in task conditions, the relation between these cortico-cerebellar structural properties and motor performance (RQ2) was evaluated in each sub-study separately.

### 2.2 Participants

All participants were in good cognitive health [Montreal Cognitive Assessment questionnaire (MoCa) ≥ 26 (Nasreddine et al., 2005)], right-handed (Edinburgh Handedness Inventory: Laterality quotient ≥ 50; Oldfield, 1971) and did not report any neuropsychiatric and/or neuromuscular disorders or contraindications for MRI (e.g., history of a major disease, brain injury, brain surgery or medication influencing the central nervous system). The study was approved by the Medical ethics committee of the KU Leuven / UZ Leuven (study number S66026) in accordance with the Declaration of Helsinki (World Medical Association, 2013). All participants gave written informed consent prior to the start of the study and received monetary compensation for their participation.

Younger and older adults did not differ significantly in height or weight. Solely in terms of physical activity (assessed using the International Physical Activity Questionnaire [IPAQ]; Craig et al., 2003) age-group differences were present, with older adults engaging in more physical activity than younger adults in one of the two sub-studies (Sub-study 2; see Table 1). A summary of these demographics can be found in Table 1.

### 2.3 Bimanual tracking task

The BTT is a motor learning task where participants have to rotate two dials with their index fingers in a certain direction and at a certain speed to follow a moving dot on the screen (Sisti et al., 2011; Zivari Adab et al., 2020). A trial consists of three phases: (1) the planning phase, consisting of the appearance of a dot and a line indicating the trajectory along which the dot will move; (2) the execution phase, starting with the dot moving along the line, and followed by participants tracking the dot by rotating the dials, while receiving concurrent feedback of their trajectory with a red line; (3) an inter-trial-interval, during which a black screen is presented. Trial difficulty was manipulated by varying the speed and direction of rotation of each dial (i.e., inter-hand frequency ratio), resulting in different task conditions.

Several task conditions were used: Line 1:1, Line 1:3, Line 3:1, and Angle 3:1. ‘Line’ and ‘Angle’ conditions differed in the existence of directional change in the dials’ rotation within a single trial. Whereas in ‘Line’ conditions, the dial rotation direction was kept consistent throughout the entire trial duration, during ‘Angle’ conditions, it shifted in the middle of the trial for the left hand. The inter-hand frequency ratio reports to the number of rotations necessary from each dial to produce a single unit of movement. In ‘1:1′ conditions, the targeted trajectory was achieved by rotating both fingers at the same speed, while in ‘1:3′ and ‘3:1′ conditions, one of the fingers needed to rotate three times faster than the other (the right and the left, respectively).

In both sub-studies, the rotation of the left index finger controlled movements along the Y-axis (clockwise [C] = upwards, counterclockwise [CC] = downwards), rotation of the right index finger controlled movements along the X-axis (C = right, CC = left).

#### 2.3.1 Performance on the task

Performance on each trial of the bimanual tracking task is estimated via the formula in the equation below. The resulting score (S; in %) equals performance (P) multiplied by a correction factor (D).

$$S=P*D\;\text{with}\;D=(1-\frac{\bar{d}}{U})$$

The performance (P) captures the percentage of unique points in the trajectory covered by the participant in the correct order (with a sampling rate of 20 Hertz), as defined by Zivari Adab et al. (2020). Performance is then multiplied by a correction factor (D) that takes into account the mean euclidean distance ( $\bar{d}$ ) between the participant’s movement and the target line along the trajectory divided by a factor U, as done in previous studies (Hehl et al., 2025; Van Malderen et al., 2025). This aims to reduce accuracy for trajectories that were correct in speed and direction, but parallel to instead of overlapping with the target trajectory. This factor U was set to 8 to optimally capture performance in a sample of younger and older adults, thereby minimizing the ceiling and floor effects in both age groups. This was done by evaluating the skewness in three similar datasets collected by our research group.

Overall motor performance (i.e., BTT score) was estimated by calculating the mean across the scores (S) on all trials during the functional imaging acquisition, for each participant.

#### 2.3.2 Familiarization session

Prior to the BTT performance inside the scanner, all participants underwent a familiarization session to get acquainted with the task. Participants in Sub-study 1 completed a fixed amount of 15 trials of the Line1:1 condition. Participants in Sub-study 2 performed conditions Line 1:1, Line 3:1 and Angle 3:1 in multiple directions, in blocks of 12 trials (resulting from presenting each condition in all four possible combinations of dial direction), until they reached an overall mean block score of at least 15%, in a minimum of two to a maximum of eight blocks.

#### 2.3.3 BTT acquisition during the MRI scan

Although the present study focuses on the dMRI data of both sub-studies, participants in both studies also participated in a fMRS (Sub-study 1) or tb-fMRI (Sub-study 2) experiment, during which the BTT was performed inside the scanner. The task performance analyzed in the present study was extracted from the BTT being performed during these acquisitions.

Each participant completed three trial conditions. In Sub-study 1, Line1:1, Line1:3 and Line3:1 conditions were included; in Sub-study 2, Line1:1, Line3:1 and Angle3:1. The exact timings of each trial differed between the two sub-studies (see Supplementary Table 1 for a detailed overview). Importantly, the trial timing and task conditions were kept constant for all participants within the same sub-study. Regarding trial order, all participants completed the trials in the same order in Sub-study 1; whereas Sub-study 2 adopted a pseudo-randomization by assigning participants to one out of six options of trial order. Sub-study 1 consisted of 48 trials in total (16 trials per condition, organized in 1 block [~11 min]) and Sub-study 2 of 45 trials (15 trials per condition, organized in 5 blocks of 3 trials per condition [~6 min]).

### 2.4 Experimental procedure

All participants underwent a screening session, a familiarization session and one or two MRI sessions. An overview of the experimental procedure is presented in Figure 1. In Sub-study 1, the screening session lasted for about 1 h and was followed immediately by the BTT familiarization session in a mock MRI scanner. The MRI session, performed on another day, consisted of acquisition of T1 weighted (T1w) and T2 weighted (T2w) anatomical images; a fMRS scan, during which participants executed the BTT task; a dMRI scan; and a resting-state functional MRI (rs-fMRI) scan.

**Figure 1:**
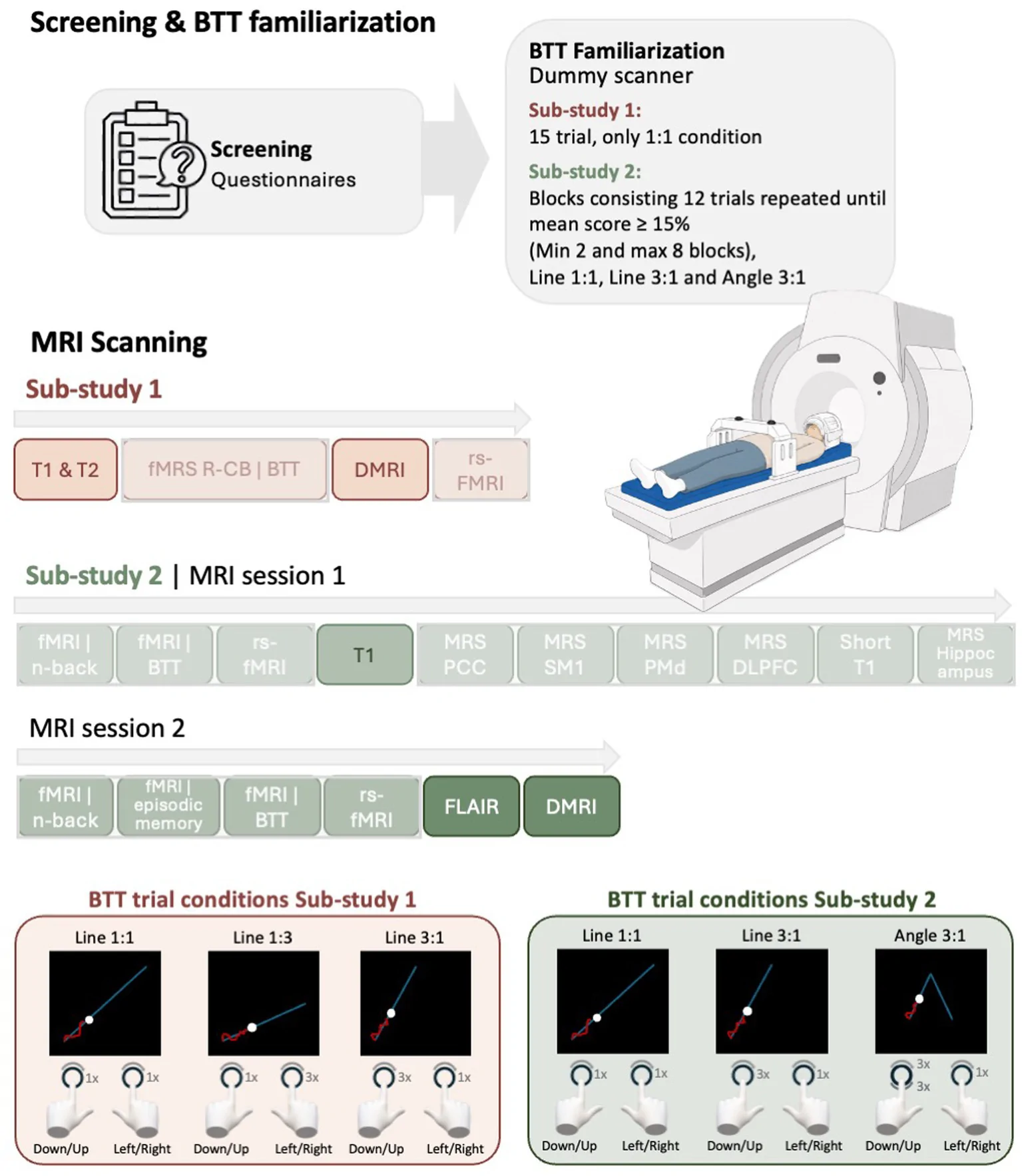
Overview of the experimental procedure and bimanual tracking task (BTT) conditions. All participants underwent a screening session, a BTT familiarization session in a mock magnetic resonance imaging (MRI) scanner, and one (Sub-study 1) or two (Sub-study 2) MRI sessions. Following the screening, participants completed a BTT familiarization protocol. In Sub-study 1, this familiarization session consisted of 15 trials of the Line 1:1 condition. In Sub-study 2, participants completed blocks of 12 trials comprising the Line 1:1, Line 3:1, and Angle 3:1 conditions until an overall mean block score of at least 15% was reached (minimum two and maximum eight blocks). Sub-study 1 included a single MRI session consisting of T1- and T2-weighted anatomical imaging, functional magnetic resonance spectroscopy (fMRS) during BTT performance, diffusion magnetic resonance imaging (dMRI), and resting-state functional magnetic resonance imaging (rs-fMRI). Sub-study 2 included two MRI sessions separated by a 2-week home-based BTT training programme. MRI session 1 consisted of task-based fMRI during the n-back and BTT tasks, rs-fMRI, T1 weighted anatomical imaging, and magnetic resonance spectroscopy (MRS) acquisitions in five regions of interest. MRI session 2 consisted of task-based fMRI during the n-back episodic memory, and BTT tasks, followed by rs-fMRI, FLAIR imaging, and dMRI. For the current manuscript, only the structural T1- and T2- weighted images / FLAIR images, as well as the dMRI data were used from each sub-study. Motor performance was estimated based on BTT performance during fMRS and BTT tb-fMRI (from Pre) acquisitions, from Sub-study 1 and Sub-study 2, respectively. The lower panels illustrate the BTT conditions included in each sub-study. In both sub-studies, the Line 1:1 and Line 3:1 conditions were performed, whereas the Line 1:3 condition was included only in Sub-study 1 and the Angle 3:1 condition only in Sub-study 2. Participants tracked a white target dot moving along a blue target line using coordinated rotations of the left and right index fingers, which controlled movement along the Y- and X-axis, respectively. BTT = bimanual tracking task; dMRI = diffusion magnetic resonance imaging; FLAIR = fluid-attenuated inversion recovery; fMRS = functional magnetic resonance spectroscopy; MRS = magnetic resonance spectroscopy; PCC = posterior cingulate cortex; PMd = dorsal premotor cortex; DLPFC = dorsolateral prefrontal cortex; rs-fMRI = resting-state functional magnetic resonance imaging; SM1 = primary sensorimotor cortex; tb-fMRI = task-based functional magnetic resonance imaging.

In Sub-study 2, screening and cognitive testing lasted between one to 3 hs, performed in one or two sessions (depending on how tired the participant felt), and also included BTT familiarization inside the same mock scanner as for Sub-study 1. For this sub-study, participants completed two MRI sessions (Pre and Post), separated by a 2-week home-based BTT training program. The Pre-session consisted of two tb-fMRI scans (with the n-back and BTT task, respectively); a T1w anatomical image; Magnetic Resonance Spectroscopy (MRS) acquisitions in five regions of interest; and a rs-fMRI scan. The Post-session included three tb-fMRI scans (n-back, episodic memory and BTT task); a dMRI scan; a rs-fMRI scan; and a fluid-attenuated inversion recovery (FLAIR) image.

For the current manuscript, only the structural T1w and T2w / FLAIR images, as well as the dMRI data were used from each sub-study. Motor performance was estimated based on BTT performance during fMRS and BTT tb-fMRI (from Pre) acquisitions, from Sub-study 1 and Sub-study 2, respectively.

### 2.5 MRI acquisition parameters

Data were acquired on a Philips Achieva dStream 3 Tesla MRI scanner (University Hospital Leuven, Gasthuisberg) with a 32-channel receive headcoil. Diffusion MRI images, encompassing the main modality of interest for this study, were collected using the exact same High Angular Resolution Diffusion Imaging (HARDI) protocol in the two sub-studies. The protocol consisted of a spin echo echo-planar imaging sequence. Data were acquired in 148 uniformly distributed directions covering b-values of 0/400/1000/2600, in respectively, 8/24/48/68 different directions. All dMRI scans were acquired with phase-encoding along the anterior–posterior axis – half acquired with an anterior–posterior phase encoding direction, and half with the opposite phase encoding (i.e., reverse-phase encoding). Anatomical images, used for preprocessing the data, consisted of T1w high-resolution anatomical images (three-dimensional gradient turbo field echo sequence) and T2w (Sub-study 1) or FLAIR images (Sub-study 2). Acquisition parameters for the dMRI and anatomical images are summarized in Supplementary Table 2.

### 2.6 Image processing

Anatomical T1w reference images were preprocessed using fMRIPrep 24.0.1 (Esteban et al., 2019; Markiewicz et al., 2024; RRID: SCR_016216) including bias field correction, skull stripping and brain tissue segmentation. Details of the fMRIPrep pipeline are reported in the Supplementary material — Section 1.3.

T2w and FLAIR images were brain extracted using hd-BET (Isensee et al., 2019; RRID: CR_014586) and co-registered to the T1w image with ANTs (Avants et al., 2011; RRID: SCR_004757). The quality of the T1w images was assessed visually and using mriqc 24.1.0 (Esteban et al., 2017; RRID: SCR_022942). Total intracranial volume (TIV) was estimated using FreeSurfer 7.4.1 (Fischl, 2012; RRID: SCR_001847) *recon-all* command, based on both the T1w and the T2w or FLAIR images, depending on their availability.

Anatomical images were furthermore used to identify and segment white matter hyperintensities (WMH) and periventricular spaces (PVS) in a semi-automatic manner: started from an initial segmentation performed with FreeSurfer 7.2.0 SamSeg (Puonti et al., 2016) based on the T1w images, which was further refined manually using FLAIR or T2w images (depending on their availability) by two researchers (SVM and MB), in direct consultation with a neuroradiologist (AR). Segmentation happened in the T1w space and voxel dimensions [i.e., (1 × 0.89 × 0.89 mm^3^) and (0.8 × 0.8 × 0.8 mm^3^)]; this was regridded to the diffusion images voxel size [i.e., (1.3 × 1.3 × 1.3 mm^3^) after preprocessing, see below] using nearest neighbor interpolation. The PVS were initially identified by ShiVai PVS-v2 (Boutinaud et al., 2021), based on the T1w images with voxel sizes of [1 × 1 × 1 mm^3^]. The resulting probability maps were binarized with a threshold set at 0.5, and regridded to the diffusion images voxel size [i.e., (1.3 × 1.3 × 1.3 mm3) after preprocessing, see below] using nearest neighbor interpolation. These were finally corrected for falsely identified PVS by subtracting the WMH map from the PVS map. The resulting PVS and WMH maps were combined to create a final lesion map and a brain mask exempt of lesions for each participant. These lesion maps were later used to exclude pathological tissue from template building and further analyses, thereby ensuring a focus on healthy aging.

Diffusion preprocessing was conducted using in-house scripts in accordance with the Mrtrix3 guidelines (MRtrix3, 2026).^2^ Preprocessing steps included denoising, Gibbs ringing correction, motion and distortion correction, N4 bias field correction and upsampling the images to a 1.3 mm isotropic resolution in that respective order, relying on FSL (Jenkinson et al., 2012; RRID: SCR_009461), ANTs (Avants et al., 2011; RRID: SCR_004757) and MRtrix3 3.0.4 (Tournier et al., 2019; RRID: CR_006971). Afterwards, diffusion images were registered to the subjects’ T1w images using ANTs (Avants et al., 2011; RRID: SCR_004757). Subsequently, a final brain mask was created using hd-BET (Isensee et al., 2019; RRID: CR_014586) and further adapted by excluding the subject-specific lesion map. Quality control of the diffusion images was done both visually and using FSL *eddy QC* (Andersson and Sotiropoulos, 2016; Bastiani et al., 2019). Participants with a clear part of the brain falling out of the field of view, and/or who were considered outliers based on presenting high absolute motion or low contrast-to-noise ratio — as identified by a value more than three standard deviations from the mean, were excluded from the analyses.

Based on the preprocessed and upsampled diffusion images, fiber orientation distribution (FOD) images were estimated using MSMT-CSD (Jeurissen et al., 2014) for each subject using study-average tissue response functions (Tournier et al., 2019; Dhollander et al., 2021). Upsampling the diffusion MRI images prior to FOD estimation and subsequent template building is aimed to improve anatomical contrast, thereby facilitating more accurate template building, registration, tractography and subsequent statistical analyses (MRtrix3, 2026; Schilling et al., 2026).^3^ Joint bias field correction and global intensity normalization of the multi-tissue compartment maps were applied to the resulting FOD images. A single study-specific white matter FOD template was generated by taking the median value of the normalized FOD images across a subset of 40 participants (10 per age group and sub-study) using the MRtrix3 *population_template* command (Tournier et al., 2019). Within each age group and sub-study, participants selected for the template had the largest extent of the brain covered in the field of view and least amount of WMH. The resulting template was used to create a template fixel mask.

Spatial normalization of subjects’ FODs to template space was subsequently achieved through the following MRtrix3 commands (Tournier et al., 2019) (1) *mrregister* to estimate a subject-to-template warp (Raffelt et al., 2011; Raffelt et al., 2012b), (2) mrtransform to warp the individual subject normalized FODs to template space without reorienting the FODs (Raffelt et al., 2012b), (3) *fod2fixel* to segment the fixels for each subject (Raffelt et al., 2012a; Smith et al., 2013), (4) *fixelreorient* to reorient the fixels using the subject-to-template warp from step 1 and (5) *fixelcorrespondence* to assign the subject fixels to the template fixels. Quality control of the spatial normalization involved visually checking the correspondence of the subjects’ fixels to the template FOD images, with the corpus callosum as reference point.

### 2.7 Tractography

Both the CPCT and the DRTT were generated using the Fun With Tracts (FWT) pipeline (Radwan et al., 2022). First, a synthesized T1w image was created for the brain template which was further segmented using FreeSurfer 6.0 (Fischl, 2012; RRID: SCR_001847) and the multi-scale brain parcellator (MSBP) (Tourbier et al., 2019). All necessary inclusion and exclusion regions of interest (ROIs) were created based on the available atlases and ROIs in the FWT pipeline. The relevant ROIs were visually checked and manually corrected if accurate segmentation of the synthesized T1w template image failed. Fiber tracking was performed using the MRtrix3 command *tckgen* with “iFOD2” (Tournier et al., 2019). Streamlines were further filtered using ScilPy 1.5 (Bore et al., 2025). An overview of the inclusion and exclusion ROIs as well as the parameters for the *tckgen* command for the CPCT and DRTT can be found in Supplementary Table 3.

A tract-specific fixel mask was defined by converting the reconstructed tracts to fixel space using the MRtrix3 command *tck2fixel* (Tournier et al., 2019). The resulting fixels were then multiplied by the whole brain fixel mask, with a tract density threshold set to a minimum of 200. The reconstructed cortico-cerebellar tracts are visualized in Figure 2.

**Figure 2:**
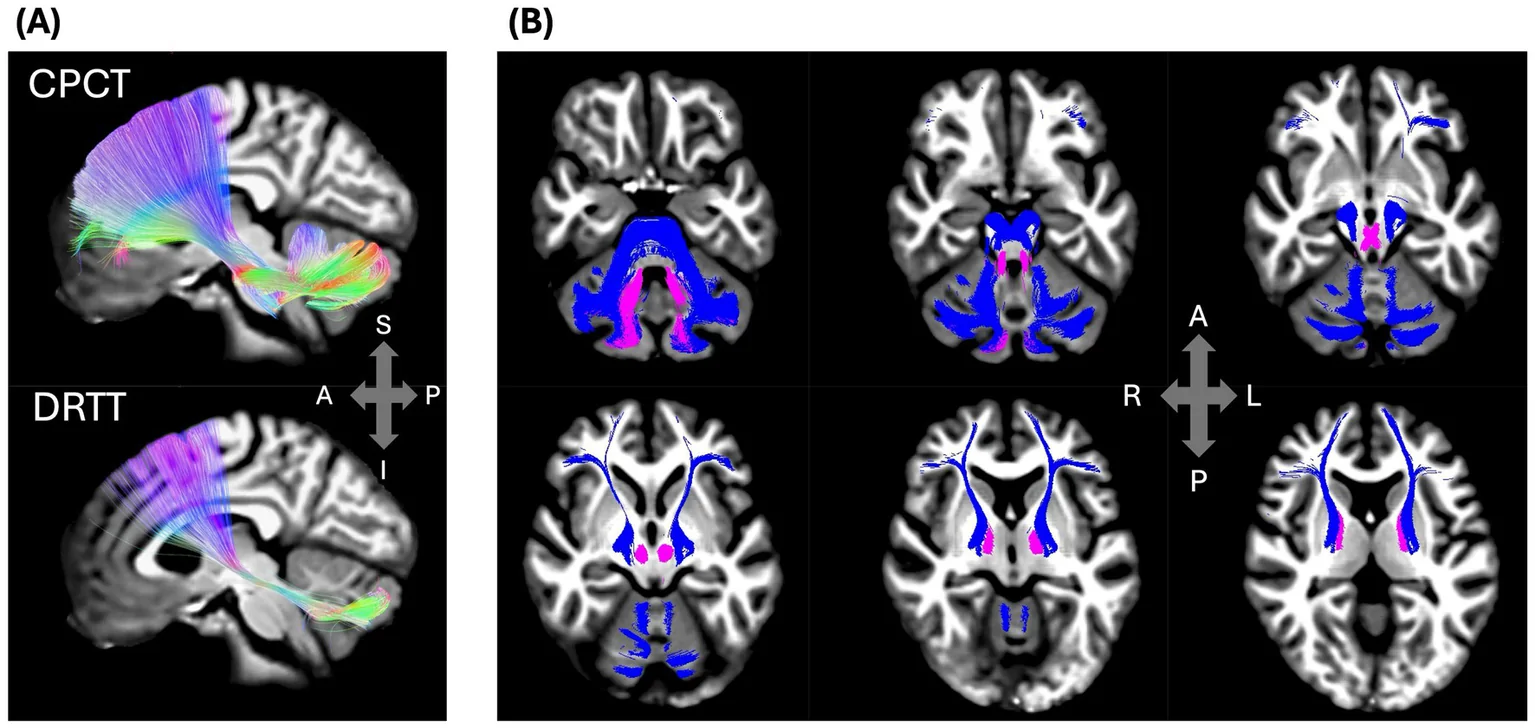
Visualization of the tractography results for the DRTT and the CPCT in study-specific template space. **(A)** Tractogram of the CPCT (top) and DRTT (bottom) in sagittal plane colored by streamline direction (anterior–posterior = green, superior–inferior = blue, left–right = red) in sagittal view. **(B)** Axial view of the DRTT in purple, and the CPCT in blue. Arrows indicate image orientation: A–P = anterior–posterior; S–I = superior–inferior; R–L = right–left. CPCT = cortico-ponto-cerebellar tract, DRTT = dentato-rubro-thalamo-cortical tract.

Although anatomically constrained tractography (ACT) is generally considered superior for whole-brain tractography (Smith et al., 2012), it performed poorly for the subcortical and cerebellar tracts examined here and was therefore not used for tract generation. Nevertheless, to evaluate whether our findings were sensitive to the absence of anatomical constraints, we conducted supplementary analyses that approximated aspects of the ACT framework by trimming tract endpoints at the boundaries of whole-brain WM and deep GM masks generated from the study-specific template using the MRtrix3 5ttgen pipeline (Tournier et al., 2019).

### 2.8 White matter-derived metrics of interest

#### 2.8.1 Theoretical background

FBA allows micro- and macrostructural properties of specific fiber populations to be disentangled. At the microstructural level, the density of the fibers within a certain spatial unit (e.g., a voxel) is captured by the apparent fiber density (FD). FD is approximately proportionally related to the total amount of intra-axonal volume under certain conditions and captures both the axonal count as well as the axonal density within the voxel (and along the fixel orientation) simultaneously (Dhollander et al., 2021). At a macrostructural level, changes in the cross-sectional area of the axonal bundles (i.e., the morphology of the tract) relative to the template image are captured by fiber cross-section (FC) (Raffelt et al., 2017). The total effect of micro- and macrostructural properties is captured by the combined fiber density and cross-section metric (FDC) (Raffelt et al., 2017; Dhollander et al., 2021). Importantly, evaluating these complementary metrics in relation to one another is essential to fully understand age-related changes in WM structure (Dhollander et al., 2021).

#### 2.8.2 Extraction of the fixel-based metrics

Whole-brain fixel-based metrics were extracted by warping subject-specific FOD images to the FOD template. FD was estimated by warping FOD images to the template space without reorientation, where estimated fixels were reoriented and assigned to the corresponding fixels in template space. FC was estimated based on the subject-to-template warped FOD image and log-transformed for further analyses (logFC). Fiber density and cross-section (FDC) metric was estimated by multiplying FD and FC (Raffelt et al., 2017; Dhollander et al., 2021). For each subject, fixels identified as falling within WMH or PVS areas were excluded from all further analyses.

A whole-brain tractogram was generated at the template level (iFOD2, 20 million fibers, minimum/maximum length = 10/250, max angle = 22.5°), and posteriorly filtered using Spherical-deconvolution Informed Filtering of Tractograms (Smith et al., 2013) to 2 million streamlines. The derived fixel-to-fixel connectivity matrix was used to smooth the resulting metrics.

### 2.9 Data analysis

Statistical analyses were performed in R 4.4.2 (R Core Team, 2024; RRID: SCR_001905) – except for the fixel-level analyses (see Section 2.9.1.2), which relied on MRtrix3 (Tournier et al., 2019). A significance level of *α* = 0.05 was used for all analyses. All models included sex as covariate of no interest; normalized total intracranial volume was included where appropriate (for models including logFC and FDC). Models were optimized using backwards selection (Babyak, 2004), while retaining covariates of no interest (sex, TIV) in the model. For linear models, normality and homoscedasticity was tested visually using the Q-Q plot and the residual-by-predicted plot as well as statistically using the Shapiro–Wilk test and the Breusch-Pagan test, respectively. Where needed, BoxCox transformations were used to ensure the models’ assumptions were met. When appropriate, post-hoc analyses were performed using the R-package *emmeans* 1.10.7 (Lenth, 2025; RRID: SCR_018734); these analyses were corrected for multiple comparisons with False Discovery Rate. Note that FD, FC and FDC are only compared per age group since they are expressed in arbitrary units and it is not meaningful to compare them between different tracts (Dhollander et al., 2021). Plots were created with ggplot2 (Wickham, 2016) and *mrview* from MRtrix3 (Tournier et al., 2019; RRID: CR_006971).

Analyses at tract level include the median tract-based values for each metric. Here, a single value, per tract (DRTT and CPCT) and participant, for each FBA-derived WM metric of interest (FD, logFC, FDC) was computed. For that, the median was first estimated across all fixels within each tract of a given cortical hemisphere (e.g., DRTT LT and DRTT RT for left and right cortical hemisphere, respectively), and later averaged across the two cortical hemispheres (i.e., the values computed for DRTT resulted from averaging the median value for all fixels DRTT LT and the median value for all fixels from DRTT RT). All tract-level analyses were repeated using the trimmed cortico-cerebellar tracts, these results are reported in the Supplementary material—Sections 2.2, 3.2.2.

Analyses at fixel level capture the fixel-specific effects in the data. Here, each fixel of the cortico-cerebellar tracts was evaluated separately. For this, fixels belonging to any tract of interest (DRTT LT, DRTT RT, CPCT LT, and CPCT RT) were combined in a cortico-cerebellar fixel mask. These results were corrected using family-wise error rate.

#### 2.9.1 Age-group differences in white matter properties of the cortico-cerebellar tracts

##### 2.9.1.1 Age-group differences in structural properties at tract level

A linear mixed model was created for each metric (FD, logFC, FDC), where age group (younger vs. older), tract (DRTT vs. CPCT), and the interaction between age group and tract were included as predictors; normalized TIV (only for the logFC and FDC metrics) and sex were included as covariates of no interest.

##### 2.9.1.2 Age-group differences in structural properties at fixel level

Fixel-specific age-group differences were evaluated using a fixel-based general linear model using the MRtrix3 *fixelcfestats* command which incorporates non-parametric permutation-based inference (Tournier et al., 2019). One model was evaluated per metric with age group as predictor and normalized TIV (only for the logFC and FDC metrics) and mean-centered dummy variable for sex as covariates of no interest.

#### 2.9.2 Relationship between the cortico-cerebellar structural properties and motor performance

The relationship between the cortico-cerebellar structural metrics and motor performance was assessed separately for each sub-study. First, the effect of age group on BTT score was evaluated. Second, the effect of the tract-specific structural metrics (at tract level) on the BTT score was evaluated.

##### 2.9.2.1 Age-group differences in BTT score

The effect of age group on BTT score was explored using a linear regression model with age group as predictor and sex as covariate of no interest, separately in each sub-study.

##### 2.9.2.2 Regression of structural properties at tract level with BTT score

The relationship between cortico-cerebellar FBA-derived metric (FD, logFC, FDC) and motor performance was assessed using analysis of covariance (ANCOVA). Six models – one for each tract (DRTT and CPCT) and each FBA-derived metric (FD, logFC and FDC) – were tested in each sub-study, with mean BTT score as dependent variable; age group, the WM metric (i.e., the tract-specific median FBA-derived metric) and their interaction as predictors; and normalized TIV (only when logFC and FDC were considered) and sex as covariates of no interest. Bonferroni correction was applied, correcting for the number of models tested (*α* < 0.05/6 = 0.0083).

## 3 Results

### 3.1 Age-group differences in white matter properties of the cortico-cerebellar tracts

#### 3.1.1 Age-group difference in structural properties at tract level

FD was significantly predicted by the interaction between tract and age group [*F* (1, 90) = 16.90, *p < 0.001*] after controlling for sex [*F* (1, 89) = 0.15, *p* = 0.703]. Post-hoc analyses revealed reduced FD in older adults compared to younger adults in the CPCT [estimate (*SE*) = −0.0084 (0.0034), *t* (108) = −2.47, *p* = 0.015], but not in the DRTT [estimate (*SE*) = 0.0003(0.0034), *t* (108) = 0.08, *p* = 0.939].

After controlling for normalized TIV [*F* (1,88) = 14.37, *p* < 0.001] and sex [*F* (1,88) = 12.85, *p* < 0.001], logFC was significantly predicted by tract [*F* (1,91) = 26.00, *p* < 0.001] and age group [*F* (1,88) = 15.78, *p* < 0.001), but not by their interaction [*F* (1,90) = 1.29, *p* = 0.259]. LogFC was lower in older adults compared to younger adults [estimate (*SE*) = −0.0421 (0.0106), *t* (88) = −3.97, *p* < 0.001].

After controlling for normalized TIV [*F* (1,88) = 9.27, *p* = 0.003] and sex [*F* (1,88) = 6.01, *p* = 0.016], FDC was also significantly predicted by tract [*F* (1,91) = 1221.97, *p* < 0.001) and age group [*F* (1,88) = 11.02, *p* = 0.001), but not by their interaction [*F* (1,90) = 1.64, *p* = 0.204). FDC was lower in older adults compared to younger adults [estimate (SE) = −0.0223 (0.0067), *t* (88) = −3.32, *p* = 0.001]. Results are visualized in Figure 3.

**Figure 3:**
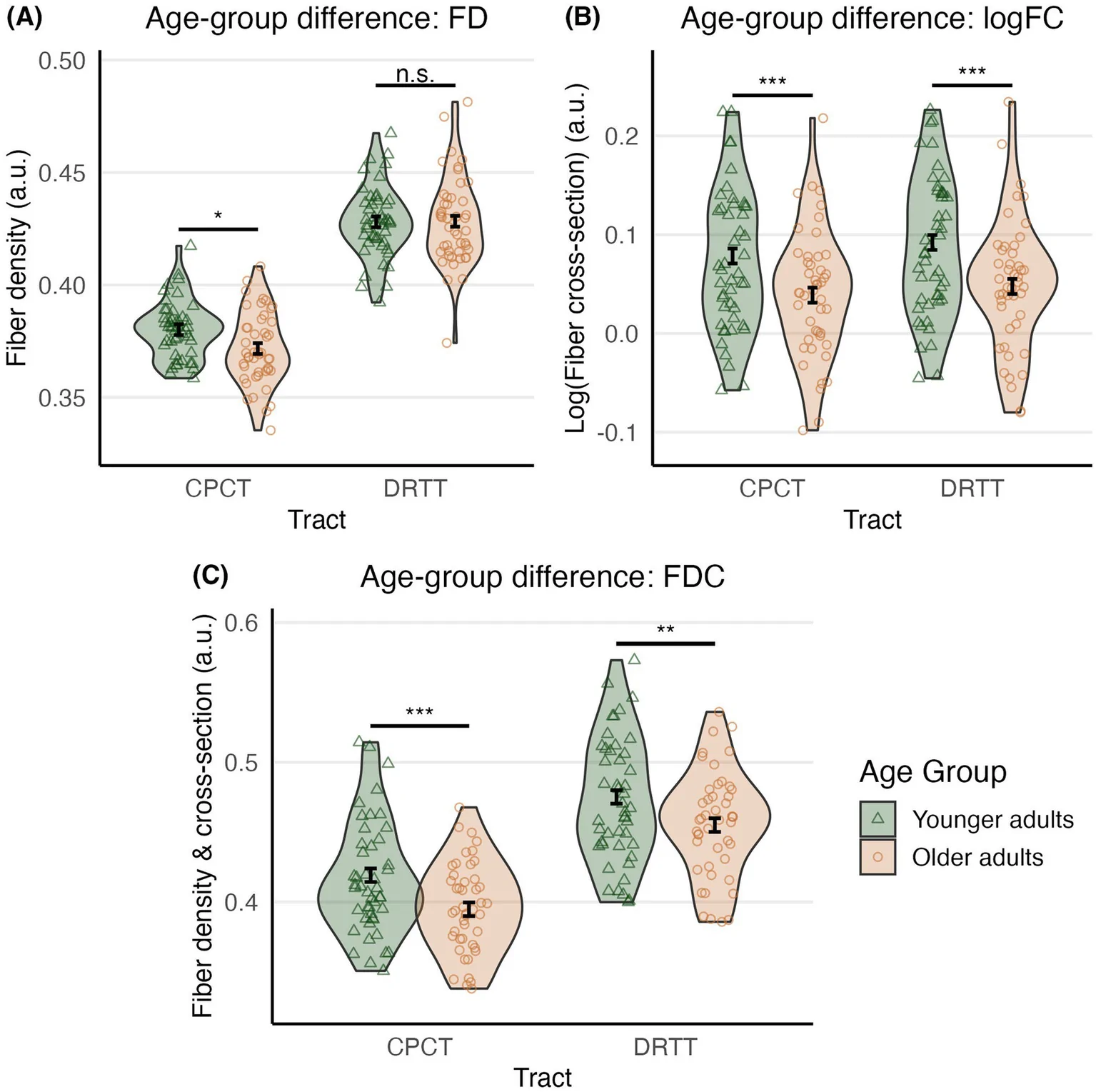
Representation of the age-group differences for each investigated structural metric of the cortico-cerebellar tracts at tract level. **(A)** Age-group differences in fiber density, **(B)** Age-group differences in logarithmic fiber cross-section, **(C)** Age-group differences in fiber density and cross-section. Younger adults are depicted in green, older adults in orange; dots and triangles represent individual data points for the younger and older adult group, respectively. Model estimated standard errors are captured by a black error bar for each tract and age group centered around the model estimated mean. Significant levels are indicated for each age group comparison per metric and tract by *: *p* < 0.05, **: *p* < 0.01, ***: *p* < 0.001. Non-significant levels are indicated with “n.s.” a.u. = arbitrary units, CPCT = cortico-ponto-cerebellar tract, DRTT = dentato-rubro-thalamo-cortical tract, FD = fiber density, FDC = fiber density and cross-section, logFC = logarithmic fiber cross-section.

#### 3.1.2 Age-group differences in structural properties at fixel level

FD, FC and FDC were lower in older adults compared to younger adults in the cerebellar hemispheres and more cranially located parts of the cortico-cerebellar tracts (see Figure 4). Interestingly, a higher FD and FDC (and to a lesser extent logFC) in older compared to younger adults was observed in the cerebellar peduncles, mostly encompassing the superior cerebellar peduncle (SCP) and the middle cerebellar peduncle (MCP). Additionally, around the midbrain there were some fixels with higher FD values combined with lower logFC values in older adults compared to younger adults, resulting in no significant group differences in FDC. Moreover, fixels from both hemispheres show similar age-group effects.

**Figure 4:**
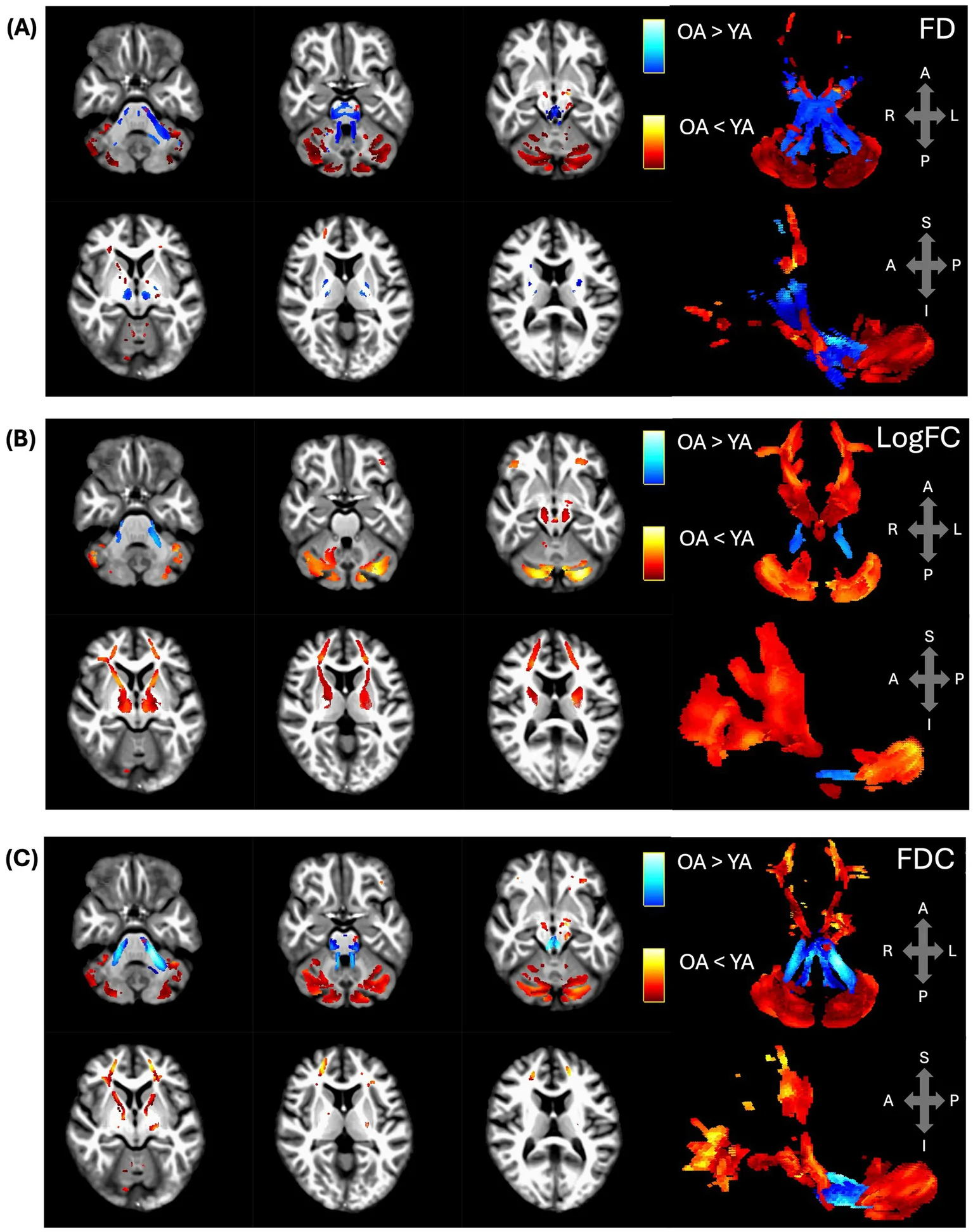
Visualization of the fixels of the cortico-cerebellar tracts exhibiting a significant age-group difference at 5% family-wise error rate corrected *p*-value for each structural metric. (A) Fiber density (FD), (B) logarithmic fiber cross-section (logFC), and (C) fiber density and cross-section (FDC). Red colors represent fixels with lower values in older compared to younger adults in the respective tract. Blue colors represent fixels with higher values in older compared to younger adults in the respective tract. Color bars represent regression coefficients (beta-values) for the group difference in arbitrary units, brighter colors indicate a larger beta. Arrows indicate the image directions: A -P = anterior -posterior, S -I = superior -inferior, R -L = right -left. CPCT = cortico-ponto-cerebellar tract, DRTT = dentato-rubro-thalamo-cortical tract, FD = fiber density, FDC = fiber density and cross-section, logFC = logarithmic fiber cross-section, OA = older adults, YA = younger adults.

### 3.2 Relation between the cortico-cerebellar structural metrics and motor performance

#### 3.2.1 Age-group differences in BTT score

A significant age-group difference in BTT score after controlling for sex was observed in Sub-study 1 [boxCox lambda = 0.358; age group: *F* (1, 42) = 42.59, *p* < 0.001; sex: *F* (1, 42) = 1.19, *p* = 0.282] and Sub-study 2 [age group: *F* (1, 42) = 50.75, *p* < 0.001; sex: *F* (1, 42) = 7.92, *p* = 0.007] (see Figure 5 and Supplementary Table 6, 7 for full model details, as well as Supplementary Figure 2 for visualization per task condition).

**Figure 5:**
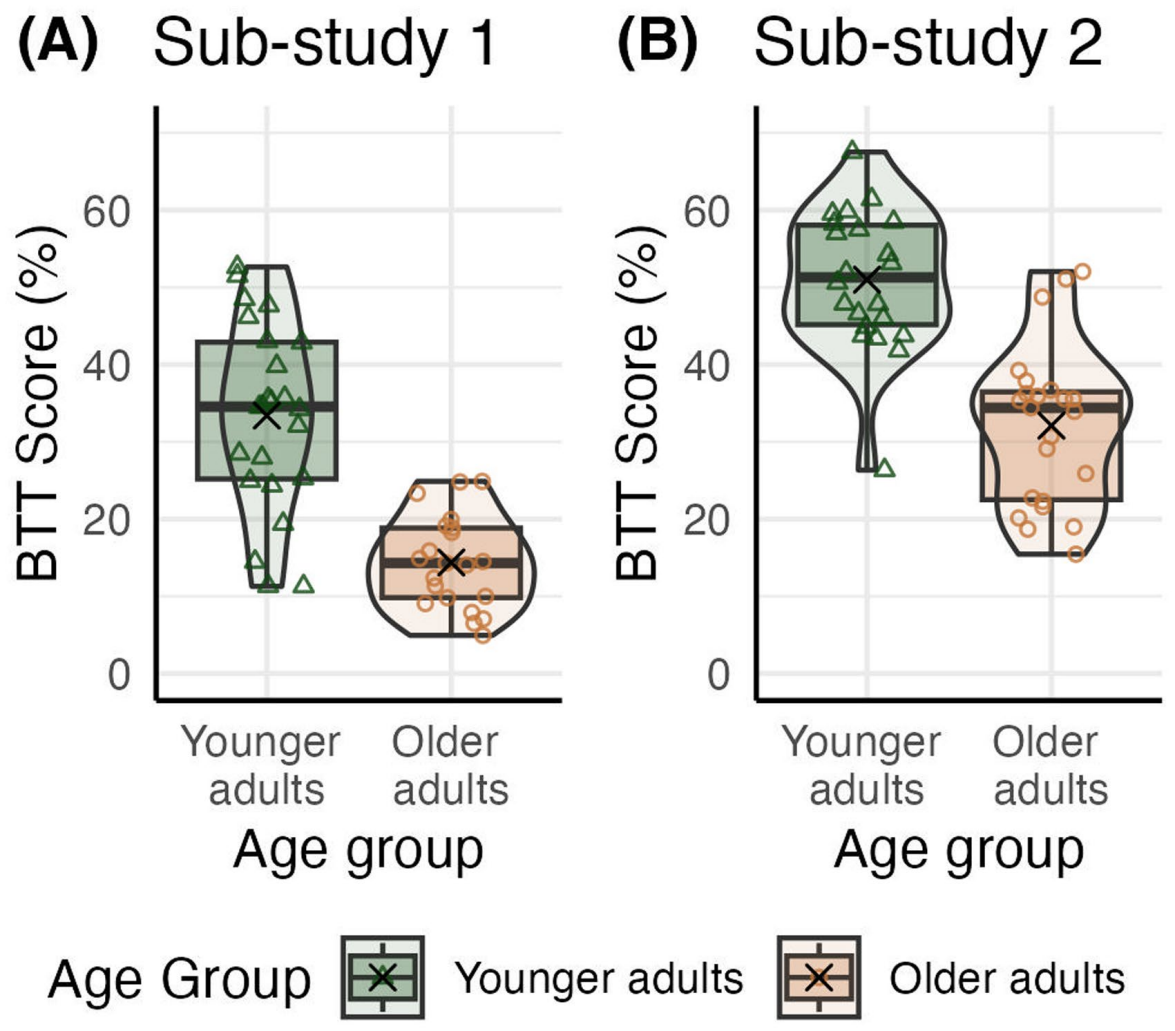
Visualization of the BTT score per age group for each sub-study. **(A)** BTT performance per age group in Sub-study 1, **(B)** BTT performance per age group in Sub-study 2. Younger adults are depicted in green, older adults in orange; dots and triangles represent individual datapoints for the younger and older adult group, respectively. Boxplots depict the median, percentile 25 and 75, and whiskers extending to 1.5 * interquartile range in both directions. The mean value is indicated by a black cross. Note that the figure shows the raw data, but analyses for Sub-Study 1 were done on the transformed data. BTT score ranges from 0 to 100%.

#### 3.2.2 Regression of structural properties at tract level with BTT score

In both sub-studies, BTT score was not significantly predicted by any of the diffusion metrics, in any of the cortico-cerebellar tracts analyzed (see Supplementary Tables 8–10). In Sub-study 2, there was a trend in the data showing logFC in the CPCT predicting BTT score [*F* (1, 40) = 4.25, *p* = 0.046] after controlling for age group [*F* (1, 40) = 31.89, *p* < 0.001], normalized TIV [*F* (1, 40) = 4.26, *p* = 0.046] and sex [*F* (1, 40) = 5.27, *p* = 0.027]. However, this trend did not survive Bonferroni correction (with *p* < 0.0083), nor was this trend observed in Sub-study 1 (BoxCox lambda = 0.346) for the logFC of the CPCT [*F* (1, 40) = 0.11, *p* = 0.740] after controlling for age group [*F* (1, 40) = 33.37, *p* < 0.001], normalized TIV [*F* (1, 40) = 0.09, *p* = 0.765] and sex [*F* (1, 40) = 1.42, *p* = 0.241]. Furthermore, evaluation of the trimmed cortico-cerebellar tracts did not show any significant correlation between the FBA-derived metrics and motor performance either. A summary of all the results can be found in Supplementary material — Section 3.2.

### 3.3 Exploratory analyses

Three post-hoc exploratory analyses were conducted. A first analysis evaluated the effect of ‘age’ on the FBA-metrics at tract-level within each age group. Here, no age-related effects were observed within the age groups (see Supplementary material—Section 4.1). A second analysis was conducted to understand potential influences of ‘sub-study’ on the tract-level age-group differences in the FBA-metrics and revealed no significant effect of ‘sub-study’ (see Supplementary material—Section 4.2). A third analysis explored the functional relevance of fixels with higher FDC in older compared to younger adults by regressing their mean values to BTT performance in the older adult group. No significant association with motor performance was observed (see Supplementary material—Section 4.3).

## 4 Discussion

The current study evaluated the effect of age-group differences in the WM structure of two primary cortico-cerebellar tracts forming reciprocal connections between the cerebellum and the cortex – the efferent DRTT and the afferent CPCT – using various FBA-derived metrics (FD, logFC or FDC). Data from two independent sub-studies revealed decreased WM structure (FDC) in older adults compared to younger adults, primarily due to macrostructural differences in the tracts at the cerebellar and cortical level. Importantly, these results were not homogeneous across all fixels within the cortico-cerebellar tracts, as some fixels around the MCP and SCP showed better structural properties in older adults compared to younger adults.

In none of the sub-studies did these age-group differences in micro- (i.e., FD) macrostructural properties (i.e., logFC) or their combination (i.e., FDC) predict motor performance.

### 4.1 Age-group differences in the white matter properties of cortico-cerebellar tracts

The first goal of the study was to evaluate age-group differences in WM properties of the DRTT and CPCT.

At tract level, an overall decrease in WM structural properties was observed in the cortico-cerebellar tracts of older adults compared to younger adults, which appeared to be largely driven by differences in macrostructure. Namely, FDC (i.e., metric capturing the combined WM micro- and macrostructural properties) and logFC (i.e., metric capturing macrostructural tract size) were consistently lower in older compared to younger adults: in the DRTT and the CPCT and also in the trimmed versions (see Supplementary Tables 4, 5 for full model details, as well as Figure 3 and Supplementary Figure 1 for visualization). On the other hand, differences in axonal microstructure (i.e., FD – composite measure of axonal density and axonal size) were much less consistent as these were only observed in the DRTT and not in the trimmed tracts. Thus, this study shows that tract-level WM structural properties of both DRTT and CPCT are decreased in older adults, with older adults showing decreased macrostructure and relatively preserved microstructure of the axons.

At fixel level, differential age-group differences were observed across different parts of the cortico-cerebellar tracts. For the majority of fixels FDC was lower in older than younger adults with both reduced axonal macrostructure (logFC) and reduced axonal microstructure (FD) in the older adult group. Interestingly, some fixels around the MCP and the SCP also showed increased axonal micro- and macrostructure in older adults compared to younger adults, resulting in increased FDC in the older adult group (see Figure 4). The pattern of local increases differed between regions. In the midbrain, higher FD occurred with lower logFC and no difference in FDC, indicating a comparable amount of fiber content distributed over a smaller cross-sectional area rather than an absolute increase in fiber content. In the cerebellar peduncles however, higher FD was accompanied by higher FDC, which cannot be explained by macrostructural change alone. These regions are among the most compact and architecturally complex of the brain, and degeneration of one fiber population against relative sparing of a crossing population has previously been shown to produce paradoxical local increases in diffusion metrics without actual increases in the spared population (Douaud et al., 2011). FD, which is officially called Apparent fiber density, is an apparent measure with known interpretational limits in such regions (Raffelt et al., 2012a; Dhollander et al., 2021). Increased partial-volume contributions at cerebrospinal fluid boundaries, given age-related posterior fossa volume loss (Raz et al., 2005), may also be a contributor.

Furthermore these findings do not provide direct evidence of functional compensation, which refers to a task-related increase in neural recruitment (Cabeza et al., 2018) and cannot be established from structural measures acquired independently of task performance; regressing the metrics of these fixels with motor performance revealed no significant relation (see Supplementary material—Section 4.3). While localized increases against a background of overall cortico-cerebellar decline could be in line with the notion of cerebellar reserve (Arleo et al., 2024), the present cross-sectional design does not test the buffering role that this account implies.

Our results are partly consistent with previous literature. On the one hand, the decreased structural properties (i.e., lower FD, logFC and FDC) in older compared to younger adults as observed for most fixels of the cortico-cerebellar tracts are in line with previous whole-brain fixel-based dMRI studies that did (e.g., Choy et al., 2020; Kelley et al., 2021; Han et al., 2023; De la Cruz et al., 2024) and did not (Zivari Adab et al., 2020) include the cerebellum. On the other hand, the local increases in FD, logFC and FDC around the MCP and SCP have only (partially) been observed by some of these studies (Han et al., 2023; De la Cruz et al., 2024). Moreover, solely the study of De la Cruz et al. (2024) reports an increased FDC around the MCP and SCP in line with our results. Several factors may account for these differential findings. Increased sensitivity of the present study to subtle age-group differences may stem from (1) the targeted focus on the cortico-cerebellar tracts and (2) the analysis approach, which controlled for WMH. In addition, discrepancies across studies may arise from differences in the age ranges examined, as well as variations in acquisition parameters, among others. To summarize, whereas overall decreases in WM properties of the cortico-cerebellar tracts of older adults are consistently found in literature (both at micro- as well as macrostructural level); the seemingly local increases in WM properties in older adults are only partially consistent with previous literature and warrant further investigation.

Overall, our results indicate that at tract level, the FDC of both DRTT and CPCT is decreased in older adults with decreased axonal macrostructure and relatively preserved axonal microstructure; however, within-tract differences in age-related effects can be observed. To better understand the nature of these locally increased FDC in older adults and its behavioral relevance, more research on age-related effects of these cortico-cerebellar tracts is needed, thereby adopting not only a tract-level but also a fixel-level analysis approach.

### 4.2 Relation between the cortico-cerebellar structural metrics and motor performance

The second goal of the study was to evaluate the importance of WM properties of the cortico-cerebellar tracts for motor performance in younger and older adults. To achieve this, data from two sub-studies were analyzed separately.

Age-group differences in motor performance were observed in both sub-studies, with older adults scoring worse than younger adults, in line with extensive literature on motor performance [for reviews see (Maes et al., 2017; Gooijers et al., 2024)]. In this study, no relation was found between the tract-based FBA-derived structural properties and performance on the BTT task. Only one of the two sub-studies showed a trend for an association between logFC of the CPCT and BTT performance, but this did not survive correction (see Supplementary Tables 8, 9). This does not support our expectations given (1) the importance of the cerebellum in the context of motor / cognitive control (e.g., Bernard, 2022; Arleo et al., 2024) and the BTT task specifically (Monteiro et al., 2017; Ferreira et al., 2026) and (2) previous studies that did show relations between motor (e.g., Zivari Adab et al., 2020) or cognitive performance (e.g., Holleran et al., 2020; Radhakrishnan et al., 2023; de la Cruz et al., 2024) and WM properties. Below several factors that might have influenced these results are discussed.

First, regressing a tract-based summary metric with motor performance is aimed to increase reliability of the metric. However, it might also have obscured more subtle associations of subparts of the tract with motor performance. In line with this, several studies evaluated tract segments and only found associations with motor or cognitive performance in certain subparts of the tracts (Zivari Adab et al., 2020; Seer et al., 2022). Similarly, Radhakrishnan et al. (2023) only found an association between specific cerebello-basal ganglia tracts and motor learning. In line with this, our analyses revealed differential age-group effects in subparts of the cortico-cerebellar tracts. Furthermore, our limited sample size might have obscured more subtle associations. Therefore, to adequately evaluate such fixel-level associations with motor performance, more research is needed with larger sample sizes.

Second, the multifaceted nature of the BTT should be considered. The BTT, and motor performance in general, encompasses multiple components such as among others, motor planning, preparation, initiation, learning, sensory integration, cognition, motor prediction and adaptation. With the cerebellum being involved in almost all these aspects (Bernard and Seidler, 2014; Arleo et al., 2024; McElroy et al., 2024), this task is highly interesting to study the importance of the cortico-cerebellar tracts for bimanual motor performance. Yet, it should be noted that motor performance in older adults originates from a complex interaction of peripheral and central factors. Specifically, age-related differences in the musculoskeletal system (e.g., joint stiffness) and sensory feedback (e.g., reduced proprioception; e.g., Hunter et al., 2016; for a review see: Wu et al., 2020; Wu et al., 2021), together with widespread structural and functional alterations in the cortex, basal ganglia and related brain networks [e.g., (Bennett et al., 2010; Goh, 2011; Bernard and Seidler, 2013; Loehrer et al., 2016; Boisgontier et al., 2018; Cassady et al., 2019; Monteiro et al., 2019); for reviews see: (Raz and Rodrigue, 2006; Seidler et al., 2010; Bhandari et al., 2016)], all contribute to motor decline. Isolating the specific contribution of the cerebellum, and particularly of the cerebro-cerebellar WM pathways, from this complex and multifactorial interplay is inherently challenging. Although reduced WM properties of these tracts may be involved in age-related motor deficits, their effect might be modest and may fall below the sensitivity of the current protocol and methods. Therefore, to fully capture the relevance of cortico-cerebellar tracts for motor performance, more research is needed trying to disentangle the different aspects of motor performance (e.g., motor adaptation, motor initiation, motor learning) and their relation to various cortical and cerebellar motor pathways.

Third, given differences in the motor task, the association between FBA-related metrics and motor performance was analyzed primarily in each sub-study separately. This resulted in a limited sample size of 45 participants per sub-study spread over the two age-groups. Indeed, a post-hoc sensitivity analysis performed using G*Power (Faul et al., 2009; RRID: SCR_013726) with *N* = 45, *α* = 0.0083 and 1-*β* = 0.8 indicates a minimum detectable Cohen’s *f*^2^ of 0.29–0.36, meaning that the study was able to detect MRI—BTT associations if at least 22.8—26.6% extra variance in the BTT scores were explained by MRI on top of Age group alone, while controlling for Sex and TIV. Consequently, smaller effects may have gone undetected, and the limited sensitivity of this study should be considered when interpreting the results for the brain-behavior relationship.

In conclusion, although previous studies have indicated the functional relevance of the cortico-cerebellar tracts in various neurological diseases such as ataxia and stroke (e.g., Schulz et al., 2015; Jang and Lee, 2021), this study cannot provide evidence for a similar importance in the context of bimanual performance in the healthy aging population.

### 4.3 Considerations and future research

A first important consideration should be made regarding the dataset. The cross-sectional nature of the current study limits our ability to attribute the observed age-group differences purely to the difference in age between the two groups. Furthermore, given differences in the motor task, data from the sub-studies could not be pooled when evaluating the brain-behavior relationship, restricting our statistical power and subsequent selection of analyses performed. Nevertheless, a strength of this study is that the same brain-behavior analyses were repeated in two independent datasets, thereby increasing the reliability of our findings.

Also, the difference in acquisition times of the dMRI data between the two Sub-studies with the dMRI data being collected after a home-based training only in Sub-study 2 might be of relevance. Literature suggests a potential effect of motor training programs on diffusion-based MRI metrics (Taubert et al., 2010; Irmen et al., 2020), which might introduce differences in the dMRI data of the two sub-studies. Supplementary material revealed that ‘Sub-study’ was not a significant predictor for the MRI-related metrics nor changed the tract-level age group differences (See Supplementary material — Section 4.2). Therefore, although the difference in acquisition times might have influenced our data, it is not expected to have affected our conclusions.

A second aspect to consider lies in the capacity of dMRI in evaluating WM structure. Although dMRI provides an indirect measure of WM properties, it enables *in vivo* approximation of axonal structure. In doing so, this study uses state-of-the art diffusion analyses techniques, specifically MSMT-CSD, which allows for the identification of crossing-fibers and the estimation of FBA-derived metrics. This approach substantially outperforms the commonly used DTI approach, thereby enabling more specific and accurate approximations of WM properties (Jeurissen et al., 2014; Dhollander et al., 2021); which is particularly important for cerebellar pathways, where the high density of fibers within a limited spatial volume leads to substantial fiber crossing within a single voxel, which cannot be accurately captured using DTI.

Third, the current study focused on specific tracts of interest to investigate the relevance of age-related differences in the cortico-cerebellar tracts for motor and cognitive function in the healthy aging population. A highly relevant research question given our limited understanding in the role of these cortico-cerebellar tracts for motor performance in aging. Therefore, future studies should aim to reproduce these analyses in a larger population of adults and attempt to relate structural alterations to motor performance. While the present study adopted a tract-specific approach based on anatomically defined white matter bundles, functionally informed parcellation frameworks such as the Multimodal White Matter Atlas (MWMA) (Zhou et al., 2026) may offer a complementary perspective for investigating age-related alterations in white matter organization and their relationship with motor performance. Additionally, given that motor performance is the result of a complex interplay between several brain networks, future research should aim to extend these analyses to the whole brain, while also focusing on structural connectivity patterns at a network level.

## 5 Conclusion

Our results indicate an overall age-related reduction in axonal properties (i.e., FDC) of the cortico-cerebellar tracts, primarily driven by decreases in macrostructural components (i.e., logFC). Notably, this reduction in structural properties was not uniform across the tracts: fixel-level analyses revealed localized increases in FD, logFC and FDC in older adults, particularly in regions around the MCP and SCP, highlighting regional heterogeneity that warrants further investigation. Despite these age-group structural differences, no evidence was found supporting a relationship between cortico-cerebellar structural properties and bimanual motor performance in younger or older adults.

## Data Availability

The datasets presented in this article are not readily available because data can be made available upon reasonable request. Requests to access the datasets should be directed to koen.cuypers@uhasselt.be.

## Footnotes

1
https://www.who.int/news-room/fact-sheets/detail/ageing-and-health

2
https://mrtrix.readthedocs.io/en/latest/fixel_based_analysis/mt_fibre_density_cross-section.html

3
https://mrtrix.readthedocs.io/en/latest/fixel_based_analysis/mt_fibre_density_cross-section.html

## Glossary

ACT: Anatomically Constrained Tractography
AD: Axial Diffusivity
BTT: Bimanual Tracking Task
C: Clockwise
CC: Counter-clockwise
CPCT: Cortico-ponto-cerebellar tract
dMRI: Diffusion-weighted Magnetic Resonance Imaging
DRTT: Dentato-rubro-thalamo-cortical tract
DTI: Diffusion Tensor Imaging
FA: Fractional Anisotropy
FBA: Fixel-Based Analysis
FC: Fiber Cross-section
FD: Fiber Density
FDC: Fiber Density and Cross-section
FLAIR: FLuid-Attenuated Inversion Recovery
fMRS: functional Magnetic Resonance Spectroscopy
FOD: Fiber Orientation Distribution
FWT: Fun with Tracts
GM: Grey Matter
HARDI: High Angular Resolution Diffusion Imaging
IQR: Inter-Quartile Range
logFC: log-transformed Fiber Cross-section
MCP: Middle Cerebellar Peduncle
MRI: Magnetic Resonance Imaging
MRS: Magnetic Resonance Spectroscopy
MSMT-CSD: Multi-Shell-Multi-Tissue Constrained Spherical Deconvolutions
PVS: Periventricular Spaces
RD: Radial Diffusivity
ROIs: Regions of Interest
rs-fMRI: resting-state functional Magnetic Resonance Imaging
SCP: Superior Cerebellar Peduncle
SD: Standard Deviation
T1w: T1 weighted
T2w: T2 weighted
tb-FMRI: task-based functional Magnetic Resonance Imaging
TIV: Total Intracranial Volume
WM: White Matter
WMH: White Matter Hyperintensities
