# Supplementary material for: Age-group differences in diffusion MRI characteristics of cortico-cerebellar tracts and their association with bimanual performance
Source: Front Aging Neurosci. 2026 Sep 17;18:1933331. doi: 10.3389/fnagi.2026.1933331 (PMC13627437; doi:10.3389/fnagi.2026.1933331)
Supplement: Supplementary file 1 [file Data_Sheet_1.pdf]

Supplementary Material

1 Methodological information

1.1 Bimanual tracking task: design parameters

	Sub-study 1		Sub-study 2	
Session	**Familiarization**	**fMRS task**	**Familiarization**	**tb-fMRI task**
Task conditions	Line1:1	Line1:1, Line1:3, Line3:1	Line1:1, Line1:3, Angle3:1	Line1:1, Line1:3, Angle3:1
Total number of trials	15	48	[24–96]	45
Number of conditions	1	3	3	3
Number of trials per condition per block	15	48	12	3
Number of blocks	1	1	[2–8] until score >15%	5
Trial order	Fixed	Fixed	Fixed	Pseudo-random
Trial duration (seconds)	Planning: 2<br>Execution: 10<br>Inter-trial-interval: 2		Planning: 1<br>Execution: 6<br>Inter-trial-interval: 1	

Supplementary Table 1. This table gives an overview of the design for the bimanual tracking task in Sub-study 1 and Sub-study 2. Abbreviations: tb = task-based, fMRI = functional Magnetic Resonance Imaging, fMRS = functional Magnetic Resonance Spectroscopy.

1.2 Magnetic Resonance Imaging acquisition parameters

	**Sub-study 1**			**Sub-study 2**		
	T1w	T2w	dMRI	T1w	FLAIR	dMRI
TE (ms)	25–27	205–218	80	46	340	80
TR (ms)	96–98	2500	4500	58–62	4800	4500
TI (ms)	-	-	-	-	1650	-
Flip angle (°)	8	90	2	8	90	2
MB factor	-	-	2	-	-	2
SENSE	-	-	2	-	-	2
Voxel size (mm^3)	1 x 0.89 x 0.89	1 x 1 x 1	2 x 2 x 2	0.8 x 0.8 x 0.8	1 x 0.98 x 0.98	2 x 2 x 2
Acquisition matrix (mm^3)	182 x 288 x 288	200 x 240 x 240	128 x 128 x 60/64	320 x 320 x 208	200 x 256 x 256	128 x 128 x 60/64

Supplementary Table 2. This table gives an overview of the acquisition parameters for the MRI scans used for each sub-study. Abbreviations: dMRI = diffusion Magnetic Resonance Imaging, FLAIR = FLuid Attenuated Inverse Recovery, ms = milliseconds, MB = multiband, SENSE = SENSE acceleration factor, T1w = T1-weighted, T2w = T2-weighted, TE = echo time, TI = inversion time, TR = repetition time.

1.3 fMRIPrep processing pipeline

Results included in this manuscript come from preprocessing performed using fMRIPrep 24.0.1 (Esteban et al., 2019; Markiewicz et al., 2024; RRID:SCR_016216), which is based on Nipype 1.8.6 (Gorgolewski et al., 2011; Markiewicz et al., 2024; RRID:SCR_002502). Each T1weighted (T1w) image was corrected for intensity non-uniformity (INU) with N4BiasFieldCorrection (Tustison et al., 2010), distributed with ANTs 2.5.1 (Avants et al., 2008; RRID:SCR_004757), and used as T1w-reference throughout the workflow. The T1w-reference was then skull-stripped with a Nipype implementation of the antsBrainExtraction.sh workflow (from ANTs), using OASIS30ANTs as target template. Brain tissue segmentation of cerebrospinal fluid (CSF), white-matter (WM) and gray-matter (GM) was performed on the brain-extracted T1w using fast (FSL (version unknown), Zhang et al., 2001; RRID:SCR_002823). Volume-based spatial normalization to one standard space (MNI152NLin2009cAsym) was performed through nonlinear registration with antsRegistration (ANTs 2.5.1), using brain-extracted versions of both T1w reference and the T1w template. The following template was selected for spatial normalization and accessed with TemplateFlow (24.2.0, Ciric et al., 2022): ICBM 152 Nonlinear Asymmetrical template version 2009c [Fonov et al. (2009), RRID:SCR_008796; TemplateFlow ID: MNI152NLin2009cAsym].
Many internal operations of fMRIPrep use Nilearn 0.10.4 (Abraham et al., 2014; RRID:SCR_001362), mostly within the functional processing workflow. For more details of the pipeline, see the section corresponding to workflows in fMRIPrep's documentation.

1.4 Tractography of the cortico-cerebellar tracts

	CPCT: left and right	DRTT: left and right
Inclusion ROIs	- Ipsilateral frontal cortex<br>- Ipsilateral anterior and posterior limb of the internal capsule (ALIC and PLIC)<br>- bilateral pons<br>- contralateral middle cerebellar peduncle (MCP)<br>- contralateral cerebellum (CB)	- ipsilateral frontal lobe<br>- ipsilateral VL thalamus<br>- ipsilateral RN<br>- contralateral nucleus dentatus
Exclusion ROIs	- contralateral superior cerebellar peduncle (SCP)<br>- ipsilateral ventrolateral (VL) thalamus<br>- ipsilateral red nucleus (RN)<br>- ipsilateral cerebellar hemisphere<br>- contralateral cerebral hemisphere	- corpus callosum<br>- contralateral cerebellar region IX<br>- ipsilateral cerebellar hemisphere<br>- contralateral cerebral hemisphere
Tractography parameters	Maximal angle: 90°<br>Length: 20 – 230 mm	Maximal angle: 45°<br>Length: 20 – 280 mm

Supplementary Table 3. Overview of inclusion ROIs, exclusion ROIs and input paramters to the MRtrix3 *tckgen* command (Tournier et al., 2019; RRID:CR_006971) to generate the CPCT and the DRTT. Abbreviations: ALIC = anterior limb of the internal capsule, CPCT = cortico-pont-cerebellar tract, DRTT = dentato-rubro-thalamo-cortical tract, mm = milimeter, PLIC = posterior limb of the internal capsule, RN = red nucleus, VL thalamus = ventrolateral thalamus.

2 Statistical results of the analyses: age-group differences in the cortico-cerebellar tracts

2.1 Age-group differences FBA-metrics at tract-level: entire tracts

2.1.1 Model parameters for the age-group comparison at tract-level: entire tracts

**FD**	Estimate (SE)	Statistic	p-value
Intercept	0.4020 (0.0016)	*F*(1, 89) = 61316.75	<.001
Tract [CPCT]	-0.0261 (0.0005)	*F*(1, 90) = 2445.72	**<.001***
Age Group [OA]	-0.0020 (0.0016)	*F*(1, 89) = 1.59	.211
Sex [female]	-0.0006 (0.0016)	*F*(1, 89) = 0.15	.703
Age Group * Tract	-0.0022 (0.0005)	*F*(1, 90) = 16.90	**<.001***
<u>FD post-hoc</u>			
CPCT [OA-YA]	-0.0084 (0.0034)	*t*(108) = -2.47	**.015***
DRTT [OA-YA]	0.0003 (0.0034)	*t*(108) = 0.08	.939
**LogFC**	Estimate (SE)	Statistic	p-value
Intercept	0.0643 (0.0052)	*F*(1, 88) = 150.23	<.001
Tract [CPCT]	-0.0056 (0.0011)	*F*(1,91) = 26.00	**<.001***
Age Group [OA]	-0.211 (0.0053)	*F*(1,88) = 15.78	**<.001***
$TIV_{Normalized}$	-0.0242 (0.0068)	*F*(1,88) = 14.37	**<.001***
Sex [female]	0.0259 (0.0068)	*F*(1,88) = 12.85	**<.001***
Age Group * Tract	-	-	-
<u>LogFC post-hoc</u>			
CPCT [OA-YA]<br>DRTT [OA-YA]	-0.0421 (0.0106)	*t*(88) = -3.97	**<.001***
**FDC**	Estimate (SE)	Statistic	p-value
Intercept	0.4361 (0.0033)	*F*(1,88) = 17284.64	<.001
Tract [CPCT]	-0.0291 (0.008)	*F*(1,91) = 1221.97	**<.001***
Age Group [OA]	-0.0111 (0.0034)	*F*(1,88) = 11.02	**.001***
$TIV_{Normalized}$	0.0132 (0.0043)	*F*(1,88) = 9.27	**.003***
Sex [female]	-0.0105 (0.0043)	*F*(1,88) = 6.01	**.016***
Age Group * Tract	-	-	-
<u>FDC post-hoc</u>			
CPCT [OA-YA]<br>DRTT [OA-YA]	-0.0223 (0.0067)	*t*(88) = -3.32	**.001***

Supplementary Table 4. Overview of the parameter estimates for the statistical models evaluating the age-group differences in the structural properties of the cortico-cerebellar tracts at tract-level. Parameter estimates are estimated using effect coding (i.e. with the average across all possible factors as reference level). Non-significant interaction effects are excluded from the model as indicated with a dash (‘-‘). The intercept term is included for completeness but its significance is not interpreted, as it tests whether the (contrast-coded) grand mean differs from zero. Abbreviations: CPCT = cortico-ponto-cerebellar tract, FD = fiber density, FDC = fiber density and cross-section , logFC = logarithm of fiber cross-section, OA = older adults, SE = standard error, YA = younger adults.

2.2 Age-group differences FBA-metrics at tract-level: trimmed tracts

2.2.1 Model parameters for the age-group comparison at tract-level: trimmed tracts

**FD**	Estimate (SE)	Statistic	p-value
Intercept	0.4706 (0.0022)	$F(1,89) = 47912.56$	<.001
Tract [CPCT]	-0.0391 (0.0007)	$F(1, 90) = 3437.30$	**<.001***
Age Group [OA]	-0.0006 (0.00214)	$F(1, 89) = 0.08$	.780
Sex [female]	-0.0010 (0.0022)	$F(1, 89) = 0.22$	.637
Age Group * Tract	-0.0020 (0.0007)	$F(1, 90) = 9.06$	**.003***
<u>FD post-hoc</u>			
CPCT [OA-YA]	-0.0052 (0.0045)	$t(106) = -1.162$	.248
DRTT [OA-YA]	0.0028 (0.0045)	$t(106) = 0.626$	.532
**LogFC**	Estimate (SE)	Statistic	p-value
Intercept	0.0684 (0.0056)	$F(1, 88) = 148.50$	<.001
Tract [CPCT]	-0.0036 (0.0012)	$F(1, 90) = 8.40$	**.005***
Age Group [OA]	-0.0202 (0.0057)	$F(1,88) = 12.72$	**<.001***
$TIV_{Normalized}$	0.0258 (0.0073)	$F(1, 88) = 12.40$	**<.001***
Sex [female]	-0.0259 (0.0072)	$F(1, 88) = 12.78$	**<.001***
Age Group * Tract	0.0034 (0.0012)	$F(1, 90) = 7.57$	**.007***
<u>logFC post-hoc</u>			
CPCT [OA-YA]	-0.0337 (0.0116)	$t(96.3) = -2.90$	**.005***
DRTT [OA-YA]	-0.0473 (0.0116)	$t(96.3) = -4.07$	**<.001***
**FDC**	Estimate (SE)	Statistic	p-value
Intercept	0.5119 (0.0040)	$F(1, 88) = 16034.77$	<.001
Tract [CPCT]	-0.0426 (0.0009)	$F(1, 91) = 2040.61$	**<.001***
Age Group [OA]	-0.0124 (0.0041)	$F(1, 88) = 9.22$	**.003***
$TIV_{Normalized}$	-0.0175 (0.0053)	$F(1, 88) = 11.00$	**.001***
Sex [female]	-0.0119 (0.0052)	$F(1, 88) = 5.18$	**.025***
Age Group * Tract	-	-	-
<u>FDC post-hoc</u>			
CPCT [OA-YA]<br>DRTT [OA-YA]	-0.0248 (0.0082)	$t(88) = -3.04$	**.003***

Supplementary Table 5. This table provides an overview of the parameter estimates for the statistical models evaluating the age-group differences in the structural properties of the cortico-cerebellar tracts at the tract-level after trimming the tracts. Parameter estimates are estimated using effect coding (i.e. with the average across all possible factors as reference level). Significance is defined at a level of 0.05 and indicated by '*' in bold. Non-significant interaction effects are excluded from the model as indicated with a dash ('-'). The intercept term is included for completeness but its significance is not interpreted, as it tests whether the (contrast-coded) grand mean differs from zero. Abbreviations: CPCT = cortico-ponto-cerebellar tract, FD = fiber density, FDC = fiber density and cross-section , logFC = logarithm of fiber cross-section, OA = older adults, SE = standard error.

2.2.2 Visualization of the age-group differences in the structural properties of cortico-cerebellar tracts: trimmed tracts

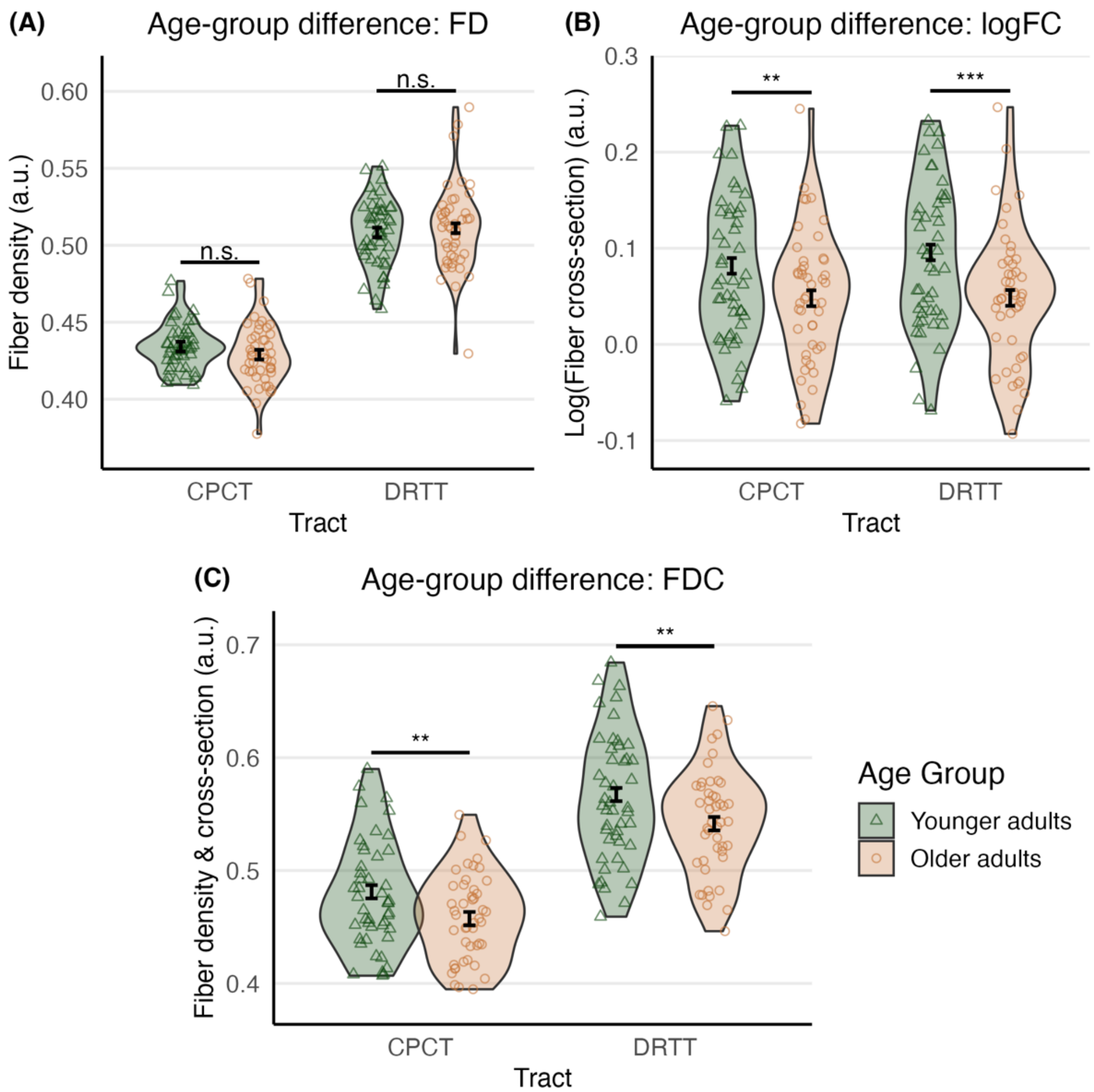

Supplementary Figure 1. Representation of the age-group differences for each investigated structural metric of the cortico-cerebellar tracts at tract-level. **(A)** age-group differences in fiber density, **(B)** age-group differences in logarithmic fiber cross-section, **(C)** age-group differences in fiber density and cross-section. Younger adults are depicted in green, older adults in orange; triangels and dots represent individual datapoints for the younger and older adult group respectively. Model estimated standard errors are captured by a black error bar for each tract and age group centered around the model estimated mean. Significant levels are indicated for each age group comparison per metric and tract by * : p <.05, ** : p <.01, *** : p <.001. Non-significant levels are indicated with 'n.s.'. Abbreviations: a.u. = arbitrary units, CPCT = cortico-ponto-cerebellar tract, DRTT = dentato-rubro-thalamo-cortical tract, FD = fiber density, FDC = fiber density and cross-section, logFC = logarithm of fiber cross-section.

3 Statistical results of the analyses: relationship between cortico-cerebellar structural metrics and motor performance

All ANOVA output below reflects the output as reproduced by the R command *Anova(model, type = 3)* from the car package (Fox and Weisberg, 2019). The intercept term is included for completeness but is not interpreted, as it tests whether the (contrast-coded) grand mean differs from zero. Tables encompassing estimation of model coefficients all implement effect-coding.

3.1 Age-group differences in the BTT score

3.1.1 Age-group differences in mean BTT score

3.1.1.1 ANOVA output: Age-group differences in BTT performance

**BTT score (%)**	**Sub-study 1 (BoxCox lambda = 0.358)**				**Sub-study 2**			
	SS	*df*	*F*	*p*	SS	*df*	*F*	*p*
(Intercept)	67,233.12	1	839.66	< .001*	78,218.96	1	970.47	< .001*
Age group	3,410.39	1	42.59	**< .001***	4,090.39	1	50.75	**< .001***
Sex	94.98	1	1.19	.282	638.66	1	7.92	**.007***
Residuals	3,363.01	42			3,385.17	42		

Supplementary Table 6. Output for the ANOVA-test evaluating the age group differences in BTT performance with as linear model: BTT score ~ Age group + Sex. Significance is defined at a level of 0.05 and indicated by '*' in bold. Abbreviations: df = degrees of freedom, *F* = F-statistic, *p* = p-value, SS = Sum of Squares.

3.1.1.2 Model coefficients: Age-group differences in BTT performance

**BTT score (%)**	**Sub-study 1 (BoxCox lambda = 0.358)**			**Sub-study 2**		
	*b**	*SE*	95% CI	*b**	*SE*	95% CI
(Intercept)	39.25	1.35	[36.52, 41.98]	41.80	1.34	[39.09, 44.50]
Age group[YA]	8.75	1.34	[6.05, 11.46]	9.54	1.34	[6.84, 12.24]
Sex	-1.48	1.35	[-4.21, 1.26]	-3.78	1.34	[-6.48, -1.07]

Supplementary Table 7. Estimated model coefficients for the linear model: BTT score ~ Age group + Sex. Abbreviations: *b** = estimated regression coefficient, CI = confidence interval, SE = standard error, YA = younger adults.

3.1.2 Age-group differences in BTT score per task condition

3.1.2.1 Vizualization of the age-group differences in BTT score per condition for each dataset

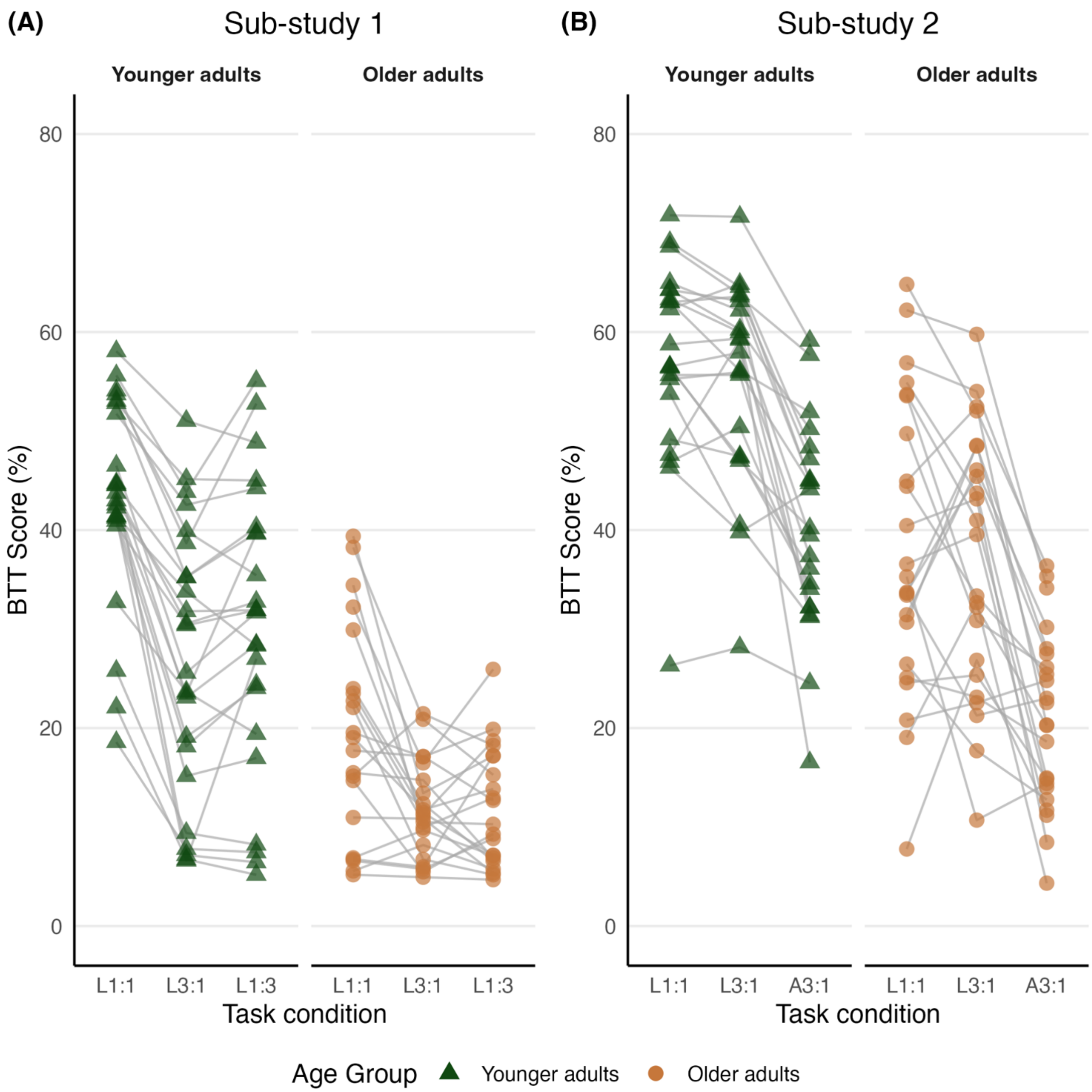

Supplementary Figure 2. Overview of the BTT scores in the two Sub-studies per age group and task condition. Younger adults are depicted in green triangles, older adults in orange dots. Data from the same participants are connected to each other. Abbreviations: L1:1 = Line 1:1, L3:1 = Line 3:1, L1:3 = Line 1:3, A3:1 = Angle 3:1.

3.2 Regression of structural properties at tract level with BTT score

3.2.1 Regression of structural properties at tract level with BTT score: entire tracts

3.2.1.1 ANOVA output: BTT score ~ FBA-metrics of the DRTT – entire tracts

**FD: DRTT**	**Sub-study 1 (BoxCox Lambda = 0.357)**				**Sub-study 2**			
Full model	SS	*df*	*F*	*p*	SS	*df*	*F*	*p*
(Intercept)	55.36	1	0.66	.421	5.85	1	0.07	.791
FD_{DRTT}	5.82	1	0.07	.794	83.26	1	1.01	.321
Age Group	13.38	1	0.16	.692	0.36	1	0.00	.948
Sex	83.32	1	0.99	.325	664.04	1	8.04	**.007***
FD_{DRTT} X Sex	2.03	1	0.02	.877	4.11	1	0.05	.825
Residuals	3,354.13	40			3,301.68	40		
Reduced model	SS	*df*	*F*	*p*	SS	*df*	*F*	*p*
(Intercept)	53.55	1	0.65	.423	9.55	1	0.12	.733
FD_{DRTT}	6.85	1	0.08	.774	79.38	1	0.98	.327
Age Group	3,415.60	1	41.73	**< .001***	4,095.59	1	50.80	**< .001***
Sex	81.35	1	0.99	.325	685.32	1	8.50	**.006***
Residuals	3,356.16	41			3,305.79	41		
**logFC: DRTT**	**Sub-study 1 (BoxCox Lambda = 0.344)**				**Sub-study 2**			
Full model	SS	*df*	*F*	*P*	SS	*df*	*F*	*p*
(Intercept)	24,389.27	1	299.86	< .001*	22,829.49	1	300.47	< .001*
$LogFC_{DRTT}$	34.77	1	0.43	.517	290.36	1	3.82	.058
Age Group	2,224.85	1	27.35	**< .001***	1,825.25	1	24.02	**< .001***
$TIV_{Normalized}$	21.35	1	0.26	.611	207.96	1	2.74	.106
Sex	204.59	1	2.52	.121	467.70	1	6.16	.018^
$LogFC_{DRTT}$ X Sex	162.83	1	2.00	.165	71.88	1	0.95	.337
Residuals	3,172.12	39			2,963.20	39		
Reduced model	SS	*df*	*F*	*p*	SS	*df*	*F*	*p*
(Intercept)	26,452.02	1	317.27	< .001*	22,759.19	1	299.95	< .001*
$LogFC_{DRTT}$	9.96	1	0.12	.731	244.51	1	3.22	.080
Age Group	2,733.00	1	32.78	**< .001***	2,614.89	1	34.46	**< .001***
$TIV_{Normalized}$	9.08	1	0.11	.743	250.35	1	3.30	.077
Sex	118.07	1	1.42	.241	460.43	1	6.07	.018^
Residuals	3,334.95	40			3,035.07	40		
**FDC: DRTT**	**Sub-study 1 (BoxCox Lambda = 0.334)**				**Sub-study 2**			
Full model	SS	*df*	*F*	*p*	SS	*df*	*F*	*p*
(Intercept)	640.88	1	7.86	.008^	35.68	1	0.46	.502
FDC_{DRTT}	24.37	1	0.30	.588	216.16	1	2.78	.104
Age Group	298.51	1	3.66	.063	151.33	1	1.94	.171
$TIV_{Normalized}$	25.09	1	0.31	.582	100.21	1	1.29	.263
Sex	202.73	1	2.49	.123	557.00	1	7.16	.011^
FDC_{DRTT} X Sex	145.75	1	1.79	.189	66.43	1	0.85	.361
Residuals	3,180.59	39			3,035.39	39		
Reduced model	SS	*df*	*F*	*P*	SS	*df*	*F*	*p*
(Intercept)	607.13	1	7.30	.010^	56.91	1	0.73	.397
FDC_{DRTT}	19.57	1	0.24	.630	177.76	1	2.29	.138
Age Group	2,979.78	1	35.83	**< .001***	3,197.47	1	41.23	**< .001***
$TIV_{Normalized}$	8.93	1	0.11	.745	170.86	1	2.20	.146
Sex	128.88	1	1.55	.220	559.57	1	7.22	.010^
Residuals	3,326.34	40			3,101.82	40		

Supplementary Table 8. Output for the ANOVA-tests evaluating the linear regression models of motor performance with BTT score. FBA-metrics are calculated from the entire left and right DRTT at tract-level. Significance was evaluated using Bonferroni correction (significance level of 0.05/6 = 0.0083): p-values < 0.05 are indicated with '^', p-values < 0.0083 are indicated with '*' and in bold. Abbreviations: df = degrees of freedom, DRTT = dentato-rubro-thalamo-cortical tract, *F* = F-statistic, FD = fiber density, FDC = fiber density and cross-section, logFC = logarithmic fiber cross-section, *p* = p-value, SS = Sum of Squares, TIV = total intracranial volume.

3.2.1.2 ANOVA output: BTT score ~ FBA-metrics of the CPCT tract – entire tracts

**FD: CPCT**	**Sub-study 1 (BoxCox Lambda = 0.360)**				**Sub-study 2**			
Full model	SS	*df*	*F*	*p*	SS	*df*	*F*	*p*
(Intercept)	32.55	1	0.39	.537	1.59	1	0.02	.890
FD_{CPCT}	9.53	1	0.11	.738	94.06	1	1.15	.290
Age Group	0.19	1	0.00	.963	5.55	1	0.07	.796
Sex	84.98	1	1.01	.320	573.97	1	7.01	.012^
FD_{CPCT} X Sex	2.30	1	0.03	.869	0.01	1	0.00	.990
Residuals	32.55	1	0.39	.537	3,277.50	40		
Reduced model	SS	*df*	*F*	*p*	SS	*df*	*F*	*p*
(Intercept)	35.08	1	0.43	.516	1.71	1	0.02	.884
FD_{CPCT}	8.64	1	0.11	.747	107.66	1	1.35	.253
Age Group	3,111.06	1	38.03	**< .001***	3,299.42	1	41.27	**< .001***
Sex	87.79	1	1.07	.306	639.14	1	8.00	**.007***
Residuals	3,354.37	41			3,277.51	41		
**logFC: CPCT**	**Sub-study 1 (BoxCox Lambda = 0.346)**				**Sub-study 2**			
Full model	SS	*df*	*F*	*P*	SS	*df*	*F*	*p*
(Intercept)	28,449.19	1	338.93	< .001*	26,222.98	1	346.17	< .001*
$LogFC_{CPCT}$	15.28	1	0.18	.672	323.75	1	4.27	.045^
Age Group	2,189.18	1	26.08	**< .001***	1,792.51	1	23.66	**< .001***
$TIV_{Normalized}$	15.96	1	0.19	.665	319.97	1	4.22	.047^
Sex	161.83	1	1.93	.173	395.75	1	5.22	.028^
$LogFC_{CPCT}$ X Sex	61.84	1	0.74	.396	10.36	1	0.14	.713
Residuals	3,273.62	39			2,954.31	39		
Reduced model	SS	*Df*	*F*	*p*	SS	*df*	*F*	*p*
(Intercept)	29,889.58	1	358.45	< .001*	26,212.77	1	353.67	< .001*
$LogFC_{CPCT}$	9.30	1	0.11	.740	314.91	1	4.25	.046^
Age Group	2,782.24	1	33.37	**< .001***	2,363.30	1	31.89	**< .001***
$TIV_{Normalized}$	7.54	1	0.09	.765	315.39	1	4.26	.046^
Sex	117.99	1	1.42	.241	390.68	1	5.27	.027^
Residuals	3,335.46	40			2,964.67	40		
**FDC: CPCT**	**Sub-study 1 (BoxCox Lambda = 0.352)**				**Sub-study 2**			
Full model	SS	*df*	*F*	*p*	SS	*df*	*F*	*p*
(Intercept)	375.92	1	4.48	.041^	32.71	1	0.41	.523
FDC_{CPCT}	2.40	1	0.03	.866	203.38	1	2.58	.116
Age Group	179.34	1	2.14	.152	78.51	1	1.00	.325
$TIV_{Normalized}$	19.87	1	0.24	.629	179.14	1	2.27	.140
Sex	143.72	1	1.71	.198	511.01	1	6.48	.015^
FDC_{CPCT} X Sex	71.46	1	0.85	.362	19.26	1	0.24	.624
Residuals	3,269.81	39			3,076.04	39		
Reduced model	SS	*df*	*F*	*P*	SS	*df*	*F*	*P*
(Intercept)	377.53	1	4.52	.040^	49.59	1	0.64	.428
FDC_{CPCT}	3.08	1	0.04	.849	184.28	1	2.38	.131
Age Group	2,713.37	1	32.48	**< .001***	2,624.29	1	33.91	**< .001***
$TIV_{Normalized}$	10.15	1	0.12	.729	196.99	1	2.55	.118
Sex	110.60	1	1.32	.257	502.35	1	6.49	.015^
Residuals	3,341.27	40			3,095.30	40		

Supplementary Table 9. Output for the ANOVA-tests evaluating the linear regression models of FBA-metrics with BTT score. FBA-metrics are calculated from the entire left and right CPCT at tract-level. Significance was evaluated using Bonferroni correction (significance level of 0.05/6 = 0.0083, in BOLD); p-values < 0.05 are indicated with '^', p-values < 0.0083 are indicated with '*' and in BOLD. Abbreviations: CPCT = cortico-ponto-cerebellar tract, df = degrees of freedom, *F* = F-statistic, FD = fiber density, FDC = fiber density and cross-section, logFC = logarithmic fiber cross-section, *p* = p-value, SS = Sum of Squares, TIV = total intracranial volume.

3.2.1.3 Model coefficients: final models BTT score ~ FBA-metrics – entire tracts

**FD: DRTT**	**Sub-study 1 (BoxCox Lambda = 0.357)**			**Sub-study 2**		
Final model	*b**	*SE*	95% CI	*b**	*SE*	95% CI
(Intercept)	28.95	35.79	[-43.33, 101.22]	10.77	31.30	[-52.44, 73.98]
FD_{DRTT}	24.26	83.90	[-145.17, 193.70]	72.08	72.64	[-74.63, 218.79]
Age Group [YA]	8.76	1.36	[6.02, 11.50]	9.55	1.34	[6.84, 12.25]
Sex [female]	-1.39	1.40	[-4.22, 1.43]	-3.94	1.35	[-6.67, -1.21]
**FD: CPCT**	**Sub-study 1 (BoxCox Lambda = 0.360)**			**Sub-study 2**		
Final model	*b**	*SE*	95% CI	*b**	*SE*	95% CI
(Intercept)	26.17	39.96	[-54.53, 106.86]	4.68	32.01	[-59.96, 69.33]
FD_{CPCT}	34.67	106.64	[-180.71, 250.04]	98.34	84.74	[-72.79, 269.47]
Age Group [YA]	8.64	1.40	[5.81, 11.47]	9.03	1.40	[6.19, 11.86]
Sex [female]	-1.43	1.38	[-4.21, 1.35]	-3.78	1.34	[-6.48, -1.08]
**logFC: DRTT**	**Sub-study 1 (BoxCox Lambda = 0.344)**			**Sub-study 2**		
Final model	*b**	*SE*	95% CI	*b**	*SE*	95% CI
(Intercept)	40.57	2.28	[35.97, 45.18]	38.65	2.23	[34.14, 43.16]
$LogFC_{DRTT}$	-8.54	24.70	[-58.46, 41.38]	50.49	28.12	[-6.36, 107.33]
Age Group [YA]	8.75	1.53	[5.66, 11.84]	8.42	1.43	[5.52, 11.32]
$TIV_{Normalized}$	-0.60	1.82	[-4.29, 3.08]	-3.78	2.08	[-7.99, 0.43]
Sex [female]	-2.19	1.84	[-5.91, 1.53]	-4.71	1.91	[-8.58, -0.85]
**logFC: CPCT**	**Sub-study 1 (BoxCox Lambda = 0.346)**			**Sub-study 2**		
Final model	*b**	*SE*	95% CI	*b**	*SE*	95% CI
(Intercept)	40.41	2.13	[36.10, 44.73]	38.61	2.05	[34.46, 42.76]
$logFC_{CPCT}$	-8.99	26.91	[-63.37, 45.40]	61.24	29.71	[1.19, 121.28]
Age Group [YA]	8.73	1.51	[5.67, 11.78]	8.15	1.44	[5.23, 11.07]
$TIV_{Normalized}$	-0.56	1.87	[-4.35, 3.22]	-4.46	2.16	[-8.83, -0.09]
Sex [female]	-2.16	1.82	[-5.83, 1.51]	-4.40	1.92	[-8.27, -0.53]
**FDC: DRTT**	**Sub-study 1 (BoxCox Lambda = 0.334)**			**Sub-study 2**		
Final model	*b**	*SE*	95% CI	*b**	*SE*	95% CI
(Intercept)	49.22	18.22	[12.40, 86.04]	15.17	17.71	[-20.62, 50.95]
FDC_{DRTT}	-18.88	38.93	[-97.56, 59.79]	57.63	38.06	[-19.30, 134.55]
Age Group [YA]	8.70	1.45	[5.76, 11.64]	8.87	1.38	[6.08, 11.66]
$TIV_{Normalized}$	-0.58	1.78	[-4.17, 3.01]	-2.92	1.97	[-6.90, 1.06]
Sex [female]	-2.29	1.84	[-6.00, 1.43]	-5.12	1.91	[-8.97, -1.27]
**FDC: CPCT**	**Sub-study 1 (BoxCox Lambda = 0.352)**			**Sub-study 2**		
Final model	*b**	*SE*	95% CI	*b**	*SE*	95% CI
(Intercept)	43.52	20.47	[2.15, 84.90]	14.34	17.91	[-21.86, 50.54]
FDC_{CPCT}	-9.66	50.36	[-111.43, 92.11]	67.70	43.87	[-20.97, 156.37]
Age Group [YA]	8.66	1.52	[5.59, 11.73]	8.51	1.46	[5.56, 11.47]
$TIV_{Normalized}$	-0.66	1.90	[-4.50, 3.18]	-3.22	2.02	[-7.30, 0.86]
Sex [female]	-2.05	1.78	[-5.64, 1.55]	-4.90	1.92	[-8.78, -1.01]

Supplementary Table 10. Estimation of the model coefficients for the final models used to evaluate the regression of FBA-related metrics with BTT score. FBA-metrics are calculated from the entire left and right CPCT or DRTT at tract-level. Abbreviations: b* = estimated regression coefficient, CI = confidence interval, CPCT = cortico-ponto-cerebellar tract, FD = fiber density, FDC = fiber density and cross-section, logFC = logarithmic fiber cross-section, SE = standard error, TIV = total intracranial volume, YA = younger adults.

3.2.2 Regression of structural properties at tract level with BTT score: trimmed tracts

3.2.2.1 ANOVA output: BTT score ~ FBA-metrics of the DRTT – trimmed tracts

**FD: DRTT**	**Sub-study 1 (BoxCox Lambda = 0.371)**				**Sub-study 2**			
Full model	SS	*df*	*F*	*p*	SS	*df*	*F*	*p*
(Intercept)	43.68	1	0.52	.473	15.91	1	0.20	.661
FD$_{DRTT}$	20.10	1	0.24	.626	99.02	1	1.22	.276
Age Group	2.01	1	0.02	.877	32.67	1	0.40	.530
Sex	74.56	1	0.90	.350	608.34	1	7.48	.009^
FD$_{DRTT}$ X Sex	15.69	1	0.19	.667	79.29	1	0.98	.329
Residuals	3,331.39	40			3,251.08	40		
Reduced model	SS	*df*	*F*	*p*	SS	*df*	*F*	*p*
(Intercept)	50.93	1	0.62	.434	54.21	1	0.67	.419
FD$_{DRTT}$	16.20	1	0.20	.658	54.80	1	0.67	.416
Age Group	3,427.79	1	41.99	**< .001***	4,121.28	1	50.74	**< .001***
Sex	83.30	1	1.02	.318	653.17	1	8.04	**.007***
Residuals	3,347.08	41			3,330.38	41	3,330.38	41
**logFC: DRTT**	**Sub-study 1 (BoxCox Lambda = 0.344)**				**Sub-study 2**			
Full model	SS	*df*	*F*	*P*	SS	*df*	*F*	*p*
(Intercept)	24,866.89	1	304.72	< .001*	26,864.63	1	346.55	< .001*
LogFC$_{DRTT}$	32.29	1	0.40	.533	188.11	1	2.43	.127
Age Group	2,214.06	1	27.13	**< .001***	2,232.20	1	28.80	**< .001***
TIV$_{Normalized}$	26.08	1	0.32	.575	146.33	1	1.89	.177
Sex	200.00	1	2.45	.126	518.90	1	6.69	.014^
LogFC$_{DRTT}$ X Sex	153.89	1	1.89	.178	126.32	1	1.63	.209
Residuals	3,182.61	39			3,023.28	39		
Reduced model	SS	*df*	*F*	*p*	SS	*df*	*F*	*p*
(Intercept)	27,250.94	1	326.70	< .001*	26,771.27	1	340.00	< .001*
LogFC$_{DRTT}$	8.42	1	0.10	.752	129.98	1	1.65	.206
Age Group	2,749.87	1	32.97	**< .001***	2,865.83	1	36.40	**< .001***
TIV$_{Normalized}$	11.12	1	0.13	.717	200.56	1	2.55	.118
Sex	115.50	1	1.38	.246	524.66	1	6.66	.014^
Residuals	3,336.50	40			3,149.60	40		
**FDC: DRTT**	**Sub-study 1 (BoxCox Lambda = 0.344)**				**Sub-study 2**			
Full model	SS	*df*	*F*	*p*	SS	*df*	*F*	*p*
(Intercept)	596.26	1	7.16	.011^	101.98	1	1.27	.267
FDC$_{DRTT}$	15.16	1	0.18	.672	136.37	1	1.69	.201
Age Group	215.77	1	2.59	.115	98.02	1	1.22	.277
TIV$_{Normalized}$	18.37	1	0.22	.641	120.05	1	1.49	.229
Sex	171.49	1	2.06	.159	583.74	1	7.25	.010^
FDC$_{DRTT}$ X Sex	88.31	1	1.06	.309	28.21	1	0.35	.557
Residuals	3,246.16	39			3,138.39	39		
Reduced model	SS	*df*	*F*	*P*	SS	*df*	*F*	*p*
(Intercept)	560.72	1	6.73	.013^	139.62	1	1.76	.192
FDC$_{DRTT}$	10.49	1	0.13	.725	112.97	1	1.43	.239
Age Group	2,919.84	1	35.03	**< .001***	3,366.07	1	42.52	**< .001***
TIV$_{Normalized}$	9.70	1	0.12	.735	164.94	1	2.08	.157
Sex	119.65	1	1.44	.238	585.45	1	7.40	.010^
Residuals	3,334.47	40			3,166.61	40		

Supplementary Table 11. Output for the ANOVA-tests evaluating the linear regression models of motor performance with BTT score. FBA-metrics are calculated from the trimmed left and right DRTT at tract-level. Significance was evaluated using Bonferroni correction (significance level of 0.05/6 = 0.0083): p-values < 0.05 are indicated with '^', p-values < 0.0083 are indicated with '*' and in bold. Abbreviations: df = degrees of freedom, DRTT = dentato-rubro-thalamo-cortical tract, *F* = F-statistic, FD = fiber density, FDC = fiber density and cross-section, logFC = logarithmic fiber cross-section, *p* = p-value, SS = Sum of Squares, TIV = total intracranial volume.

3.2.2.2 ANOVA output: BTT score ~ FBA-metrics of the CPCT tract – trimmed tracts

**FD: CPCT**	**Sub-study 1 (BoxCox Lambda = 0.366)**				**Sub-study 2**			
Full model	SS	*df*	*F*	*p*	SS	*df*	*F*	*p*
(Intercept)	44.71	1	0.54	.469	9.40	1	0.11	.738
FD$_{CPCT}$	6.80	1	0.08	.777	65.06	1	0.78	.381
Age Group	5.55	1	0.07	.798	1.86	1	0.02	.882
Sex	75.43	1	0.90	.348	545.91	1	6.58	.014^
FD$_{CPCT}$ X Sex	20.00	1	0.24	.627	14.29	1	0.17	.680
Residuals	3,339.31	40			3,319.51	40		
Reduced model	SS	*df*	*F*	*p*	SS	*df*	*F*	*P*
(Intercept)	55.93	1	0.68	.413	24.15	1	0.30	.589
FD$_{CPCT}$	3.78	1	0.05	.831	51.38	1	0.63	.431
Age Group	3,386.25	1	41.33	**< .001***	3,709.71	1	45.62	**< .001***
Sex	85.69	1	1.05	.312	632.67	1	7.78	.008^
Residuals	3,359.32	41			3,333.80	41		
**logFC: CPCT**	**Sub-study 1 (BoxCox Lambda = 0.343)**				**Sub-study 2**			
Full model	SS	*df*	*F*	*P*	SS	*df*	*F*	*p*
(Intercept)	27,223.76	1	325.19	< .001*	24,770.51	1	312.68	< .001*
LogFC$_{CPCT}$	18.03	1	0.22	.645	187.92	1	2.37	.132
Age Group	2,160.52	1	25.81	**< .001***	2,040.52	1	25.76	**< .001***
TIV$_{Normalized}$	16.06	1	0.19	.664	237.33	1	3.00	.091
Sex	165.36	1	1.98	.168	433.10	1	5.47	.025^
LogFC$_{CPCT}$ X Sex	72.31	1	0.86	.358	18.97	1	0.24	.627
Residuals	3,264.95	39			3,089.56	39		
Reduced model	SS	*df*	*F*	*p*	SS	*df*	*F*	*p*
(Intercept)	28,782.05	1	344.98	< .001*	24,785.63	1	318.94	< .001*
LogFC$_{CPCT}$	7.71	1	0.09	.763	171.04	1	2.20	.146
Age Group	2,853.71	1	34.20	**< .001***	2,895.13	1	37.25	**< .001***
TIV$_{Normalized}$	8.37	1	0.10	.753	236.36	1	3.04	.089
Sex	116.03	1	1.39	.245	427.86	1	5.51	.024^
Residuals	3,337.26	40			3,108.54	40		
**FDC: CPCT**	**Sub-study 1 (BoxCox Lambda = 0.349)**				**Sub-study 2**			
Full model	SS	*df*	*F*	*p*	SS	*df*	*F*	*p*
(Intercept)	490.40	1	5.85	.020^	88.39	1	1.08	.304
FDC$_{CPCT}$	15.47	1	0.18	.670	96.23	1	1.18	.284
Age Group	171.65	1	2.05	.160	84.70	1	1.04	.314
TIV$_{Normalized}$	9.38	1	0.11	.740	149.91	1	1.84	.183
Sex	156.65	1	1.87	.179	543.97	1	6.67	.014^
FDC$_{CPCT}$ X Sex	61.69	1	0.74	.396	20.10	1	0.25	.622
Residuals	3,267.68	39			3,181.16	39		
Reduced model	SS	*df*	*F*	*P*	SS	*df*	*F*	*p*
(Intercept)	483.97	1	5.81	.021^	124.99	1	1.56	.219
FDC$_{CPCT}$	15.19	1	0.18	.672	78.33	1	0.98	.328
Age Group	2,881.51	1	34.62	< .001*	3,040.63	1	37.99	**< .001***
TIV$_{Normalized}$	4.18	1	0.05	.824	163.64	1	2.04	.161
Sex	124.17	1	1.49	.229	542.21	1	6.78	.013^
Residuals	3,329.37	40			3,201.25	40		

Supplementary Table 12. Output for the ANOVA-tests evaluating the linear regression models of FBA-metrics with BTT score. FBA-metrics are calculated from the trimmed left and right CPCT at tract-level. Significance was evaluated using Bonferroni correction (significance level of 0.05/6 = 0.0083, in BOLD); p-values < 0.05 are indicated with '^', p-values < 0.0083 are indicated with '*' and in BOLD. Abbreviations: CPCT = cortico-ponto-cerebellar tract, df = degrees of freedom, *F* = F-statistic, FD = fiber density, FDC = fiber density and cross-section, logFC = logarithmic fiber cross-section, *p* = p-value, SS = Sum of Squares, TIV = total intracranial volume.

3.2.2.3 Model coefficients: final models BTT score ~ FBA-metrics – trimmed tracts

**FD: DRTT**	**Sub-study 1 (BoxCox Lambda = 0.371)**			**Sub-study 2**		
Final model	*b**	*SE*	95% CI	*b**	*SE*	95% CI
(Intercept)	24.73	31.31	[-38.50, 87.96]	20.86	25.53	[-30.70, 72.42]
FD_{DRTT}	27.34	61.38	[-96.61, 151.29]	41.04	49.97	[-59.87, 141.95]
Age Group [YA]	8.82	1.36	[6.07, 11.57]	9.58	1.35	[6.87, 12.30]
Sex [female]	-1.39	1.38	[-4.18, 1.39]	-3.82	1.35	[-6.55, -1.10]
**FD: CPCT**	**Sub-study 1 (BoxCox Lambda = 0.366)**			**Sub-study 2**		
Final model	*b**	*SE*	95% CI	*b**	*SE*	95% CI
(Intercept)	30.88	37.38	[-44.60, 106.36]	17.01	31.21	[-46.02, 80.04]
FD_{CPCT}	18.61	86.60	[-156.27, 193.49]	57.36	72.16	[-88.37, 203.10]
Age Group [YA]	8.74	1.36	[5.99, 11.48]	9.30	1.38	[6.52, 12.08]
Sex [female]	-1.42	1.39	[-4.23, 1.39]	-3.76	1.35	[-6.48, -1.04]
**logFC: DRTT**	**Sub-study 1 (BoxCox Lambda = 0.344)**			**Sub-study 2**		
Final model	*b**	*SE*	95% CI	*b**	*SE*	95% CI
(Intercept)	40.51	2.24	[35.98, 45.04]	39.72	2.15	[35.37, 44.08]
$LogFC_{DRTT}$	-7.25	22.81	[-53.35, 38.86]	33.59	26.14	[-19.25, 86.43]
Age Group [YA]	8.73	1.52	[5.66, 11.80]	8.75	1.45	[5.82, 11.68]
$TIV_{Normalized}$	-0.65	1.79	[-4.27, 2.96]	-3.41	2.14	[-7.73, 0.91]
Sex [female]	-2.18	1.85	[-5.92, 1.56]	-5.00	1.94	[-8.91, -1.08]
**logFC: CPCT**	**Sub-study 1 (BoxCox Lambda = 0.343)**			**Sub-study 2**		
Final model	*b**	*SE*	95% CI	*b**	*SE*	95% CI
(Intercept)	40.50	2.18	[36.09, 44.90]	39.29	2.20	[34.84, 43.74]
$logFC_{CPCT}$	-7.69	25.28	[-58.78, 43.41]	43.69	29.45	[-15.83, 103.20]
Age Group [YA]	8.69	1.49	[5.68, 11.69]	8.70	1.42	[5.82, 11.57]
$TIV_{Normalized}$	-0.59	1.86	[-4.36, 3.18]	-3.84	2.20	[-8.29, 0.61]
Sex [female]	-2.14	1.81	[-5.80, 1.52]	-4.62	1.97	[-8.60, -0.64]
**FDC: DRTT**	**Sub-study 1 (BoxCox Lambda = 0.344)**			**Sub-study 2**		
Final model	*b**	*SE*	95% CI	*b**	*SE*	95% CI
(Intercept)	46.29	17.85	[10.22, 82.36]	22.09	16.64	[-11.53, 55.72]
FDC_{DRTT}	-11.30	31.84	[-75.64, 53.05]	36.03	30.16	[-24.92, 96.98]
Age Group [YA]	8.68	1.47	[5.72, 11.65]	9.03	1.39	[6.23, 11.83]
$TIV_{Normalized}$	-0.62	1.81	[-4.26, 3.03]	-2.91	2.02	[-6.99, 1.17]
Sex [female]	-2.16	1.80	[-5.80, 1.48]	-5.23	1.92	[-9.11, -1.34]
**FDC: CPCT**	**Sub-study 1 (BoxCox Lambda = 0.349)**			**Sub-study 2**		
Final model	*b**	*SE*	95% CI	*b**	*SE*	95% CI
(Intercept)	48.28	20.02	[7.81, 88.74]	23.41	18.74	[-14.45, 61.28]
FDC_{CPCT}	-18.17	42.55	[-104.17, 67.82]	39.52	39.95	[-41.22, 120.25]
Age Group [YA]	8.74	1.49	[5.74, 11.75]	8.94	1.45	[6.01, 11.88]
$TIV_{Normalized}$	-0.43	1.94	[-4.35, 3.48]	-2.99	2.09	[-7.21, 1.24]
Sex [female]	-2.17	1.78	[-5.76, 1.42]	-5.08	1.95	[-9.03, -1.14]

Supplementary Table 13. Estimation of the model coefficients for the final models used to evaluate the regression of FBA-related metrics with BTT score. FBA-metrics are calculated from the trimmed left and right CPCT or DRTT at tract-level. Abbreviations: b* = estimated regression coefficient, CI = confidence interval, CPCT = cortico-ponto-cerebellar tract, FD = fiber density, FDC = fiber density and cross-section, logFC = logarithmic fiber cross-section, SE = standard error, TIV = total intracranial volume, YA = younger adults.

4 Exploratory analyses

4.1 Age-related effects on the FBA-metrics at tract-level within each age group: entire tracts

A post-hoc exploratory analysis evaluated the age-related effects at tract-level within each age group separately. In doing so, a linear mixed model was created per age group (younger adults, older adults) and FBA-related metric (FD, logFC, FDC) with age, tract (DRTT vs. CPCT), and the interaction between age and tract as predictors; normalized TIV (only for the logFC and FDC metrics) and sex were included as covariates of no interest.

4.1.1 Model parameters for the age-related effects on the FBA-metrics within each age group

	Younger adults			Older adults		
**Fiber density**	Estimate (SE)	Statistic	p-value	Estimate (SE)	Statistic	p-value
Intercept	0.4070 (0.0097)	$F(1, 43) = 1757.7900$	<.001	0.3933 (0.0398)	$F(1, 43) = 97.6408$	<.001
Tract [CPCT]	-0.0240 (0.0008)	$F(1, 45) = 834.3676$	**<.001***	-0.0283 (0.0007)	$F(1, 45) = 1889.1716$	**<.001***
Age	-0.0001 (0.0003)	$F(1, 43) = 0.0844$	.773	0.0001 (0.0006)	$F(1, 43) = 0.0262$	.872
Sex [female]	-0.0031 (0.0019)	$F(1, 43) = 2.4726$	.123	0.0019 (0.0027)	$F(1, 43) = 0.4883$	.488
Age X Tract	-	-	-	-	-	-
**Log(Fiber cross-section)**						
Intercept	0.0966 (0.0321)	$F(1, 42) = 9.0567$	.004	0.2199 (0.1187)	$F(1,42) = 3.4310$	.071
Tract [CPCT]	-0.0069 (0.0014)	$F(1,45) = 22.8810$	**<.001***	-0.0044 (0.0017)	$F(1,45) = 6.8694$	**.012***
Age	-0.0004 (0.0011)	$F(1,42) = 0.1142$	.737	-0.0026 (0.0017)	$F(1,42) = 2.3155$	.136
$TIV_{Normalized}$	0.0201 (0.0077)	$F(1,42) = 6.7993$	**0.013***	0.0348 (0.0113)	$F(1,42) = 9.4533$	**.004***
Sex [female]	-0.0415 (0.0078)	$F(1,42) = 28.4940$	**<.001***	-0.0063 (0.0110)	$F(1,42) = 0.3230$	.572
Age X Tract	-	-	-	-	-	-
**Fiber density and cross-section**						
Intercept	0.4516 (0.0218)	$F(1,42) = 428.9981$	<.001	0.5228 (0.0716)	$F(1,42) = 53.2767$	<.001
Tract [CPCT]	-0.0280 (0.0013)	$F(1,45) = 499.2657$	**<.001***	-0.0301 (0.0011)	$F(1,45) = 772.2313$	**<.001***
Age [OA]	-0.0001 (0.0008)	$F(1,42) = 0.0268$	.871	-0.0014 (0.0010)	$F(1,42) = 1.9238$	.173
$TIV_{Normalized}$	0.0132 (0.0052)	$F(1,42) = 6.3610$	**.016***	0.0136 (0.0068)	$F(1,42) = 3.9880$	.052
Sex [female]	-0.0202 (0.0053)	$F(1,42) = 14.5548$	**<.001***	-0.0015 (0.0066)	$F(1,42) = 0.0522$	.820
Age X Tract	-	-	-	-	-	-

Supplementary Table 14. Overview of the parameter estimates for the statistical models evaluating the age-related differences in the structural properties of the cortico-cerebellar tracts at tract-level per age group. Parameter estimates are estimated using effect coding (i.e. with the average across all possible factors as reference level). Non-significant interaction effects are excluded from the model as indicated with a dash ('-'). The intercept term is included for completeness, but its significance is not interpreted, as it tests whether the (contrast-coded) grand mean differs from zero. Abbreviations: CPCT = cortico-ponto-cerebellar tract, FD = fiber density, FDC = fiber density and cross-section , logFC = logarithm of fiber cross-section, OA = older adults, SE = standard error, YA = younger adults.

4.2 Effect of sub-study on age-group differences in FBA-metrics at tract-level: entire tracts

The effect of sub-study on the age-group differences in FBA-related metrics at tract-level was explored by adding 'sub-study' as a predictor to the linear mixed models for each metric (FD, logFC, FDC), where age group (younger vs. older), tract (DRTT vs. CPCT), and the interaction between age group and tract were included as predictors; normalized TIV (only for the logFC and FDC metrics) and sex were included as covariates of no interest.

4.2.1 Model parameters for the effect of sub-study on age-group differences in FBA-metrics

**FD**	Estimate (SE)	Statistic	p-value
Intercept	0.4020 (0.0016)	$F(1, 88) = 61928.51$	<.001
Tract [CPCT]	-0.0261 (0.0005)	$F(1, 90) = 2445.95$	**<.001***
Age Group [OA]	-0.0021 (0.0016)	$F(1, 88) = 1.75$	.189
Sex [female]	-0.0005 (0.0016)	$F(1, 88) = 0.10$	.747
Sub-study [SS1]	-0.0022 (0.0016)	$F(1, 88) = 1.90$	.171
Age Group * Tract	-0.0022 (0.0005)	$F(1, 90) = 16.91$	**<.001***
FD post-hoc			
CPCT [OA-YA]	-0.0086 (0.0034)	$t(107) = -2.54$	**0.013***
DRTT [OA-YA]	0.0001 (0.0034)	$t(107) = 0.02$	0.984
**LogFC**	Estimate (SE)	Statistic	p-value
Intercept	0.0642 (0.0052)	$F(1, 87) = 153.07$	<.001
Tract [CPCT]	-0.0056 (0.0011)	$F(1,91) = 26.00$	**<.001***
Age Group [OA]	-0.0210 (0.0052)	$F(1,87) = 16.11$	**<.001***
$TIV_{Normalized}$	0.0285 (0.0069)	$F(1,87) = 16.98$	**<.001***
Sex [female]	-0.0229 (0.0067)	$F(1,87) = 11.67$	**.001***
Sub-study [SS1]	0.0091 (0.0053)	$F(1,87) = 2.98$	.088
Age Group * Tract	-	-	-
LogFC post-hoc			
CPCT [OA-YA]<br>DRTT [OA-YA]	-0.0421 (0.0105)	$t(87) = -4.014$	**<.001***
**FDC**	Estimate (SE)	Statistic	p-value
Intercept	0.4361 (0.0033)	$F(1,87) = 17189.17$	<.001
Tract [CPCT]	-0.0291 (0.008)	$F(1,91) = 1221.97$	**<.001***
Age Group [OA]	-0.0111 (0.0034)	$F(1,87) = 10.95$	**.001***
$TIV_{Normalized}$	0.0139 (0.0044)	$F(1,87) = 9.76$	**.002***
Sex [female]	-0.0101 (0.0043)	$F(1,87) = 5.53$	**.021***
Sub-study [SS1]	0.0025 (0.0034)	$F(1,87) = 0.54$	.456
Age Group * Tract	-	-	-
FDC post-hoc			
CPCT [OA-YA]<br>DRTT [OA-YA]	-0.0223 (0.0067)	$t(87) = -3.31$	**0.001***

Supplementary Table 15. This table provides an overview of the parameter estimates for the statistical models evaluating the age-group differences in the structural properties of the cortico-cerebellar tracts at the tract-level when controlling for sub-study. Parameter estimates are estimated using effect coding (i.e. with the average across all possible factors as reference level). Significance is defined at a level of 0.05 and indicated by '*' in bold. Non-significant interaction effects are excluded from the model as indicated with a dash ('-'). The intercept term is included for completeness but its significance is not interpreted, as it tests whether the (contrast-coded) grand mean differs from zero. Abbreviations: CPCT = cortico-ponto-cerebellar tract, FD = fiber density, FDC = fiber density and cross-section , logFC = logarithm of fiber cross-section, OA = older adults, SE = standard error, SS1 = Sub-study 1.

4.3 Regression of fixels where FDC older > younger adults with BTT score in older adults

An extra analysis was conducted to further explore the functional relevance of the fixels with higher FDC in older adults versus younger adults. First, a summary value per metric (FD, logFC, FDC) was extracted per participant by averaging the values within a participant over all fixels for which older adults had a significantly higher FDC than younger adults. These summary values were regressed with BTT performance in the older adults group after controlling for Sex and normalized TIV where necessary. When normality of the residuals was not met, further models were explored with mean centered age added to the model.

4.3.1 ANOVA output: BTT score ~ FBA-metrics fixels OA>YA

**FD: OA > YA**	**Sub-study 1**				**Sub-study 2**			
	SS	*df*	*F*	*p*	SS	*df*	*F*	*p*
(Intercept)	121.38	1	3.82	.066	5.35	1	0.06	.816
$FD_{Fixels(OA>YA)}$	57.96	1	1.82	.194	29.89	1	0.31	.583
Sex	82.73	1	2.60	.124	402.21	1	4.18	.054
$Age_{mean\ centered}$	-	-	-	-	-	-	-	-
Residuals	572.37	18			1,923.26	20		
**logFC: OA > YA**	**Sub-study 1**				**Sub-study 2**			
	SS	*df*	*F*	*p*	SS	*df*	*F*	*P*
(Intercept)	2,933.71	1	79.55	< .001*	17,908.21	1	236.17	< .001*
$LogFC_{Fixels(OA>YA)}$	3.42	1	0.09	.765	0.67	1	0.01	.926
$TIV_{Normalized}$	1.32	1	0.04	.852	25.54	1	0.34	.569
Sex	48.94	1	1.33	.265	454.46	1	5.99	.025^
$Age_{mean\ centered}$	-	-	-	-	370.56	1	4.89	.040^
Residuals	626.91	17			1,364.89	18		
**FDC: OA > YA**	**Sub-study 1**				**Sub-study 2**			
	SS	*df*	*F*	*P*	SS	*df*	*F*	*p*
(Intercept)	148.11	1	4.16	.057	76.63	1	1.03	.325
$FDC_{Fixels(OA>YA)}$	25.03	1	0.70	.413	20.83	1	0.28	.604
$TIV_{Normalized}$	5.35	1	0.15	.703	32.66	1	0.44	.517
Sex	48.00	1	1.35	.262	423.31	1	5.67	.029^
$Age_{mean\ centered}$	-	-	-	-	423.66	1	5.67	.028^
Residuals	605.30	17			1,344.73	18		

Supplementary Table 16. Output for the ANOVA-tests evaluating the linear regression models of FBA-metrics with BTT score in the older adults. FBA-metrics are calculated from the fixels that showed higher FDC values in older compared to younger adults. Covariates that were not included in

the initial model are indicated with a dash ('-'). Significance was evaluated using a significance level of 0.05/3 = 0.0167, in BOLD); p-values < 0.05 are indicated with '^', p-values < 0.0167 are indicated with '*' and in BOLD. Abbreviations: CPCT = cortico-ponto-cerebellar tract, df = degrees of freedom, *F* = F-statistic, FD = fiber density, FDC = fiber density and cross-section, logFC = logarithmic fiber cross-section, *p* = p-value, SS = Sum of Squares, TIV = total intracranial volume.

4.3.1.1 Model coefficients: final models BTT score ~ FBA-metrics – fixels OA>YA

**FD: OA > YA**	**Sub-study 1**			**Sub-study 2**		
	*b**	*SE*	95% CI	*b**	*SE*	95% CI
(Intercept)	47.98	24.56	[-3.61, 99.58]	9.60	40.71	[-75.33, 94.53]
$FD_{Fixels(OA>YA)}$	-47.44	35.14	[-121.26, 26.38]	32.92	59.06	[-90.27, 156.11]
Sex [female]	-2.04	1.27	[-4.71, 0.62]	-4.23	2.07	[-8.55, 0.08]
$Age_{mean\ centered}$	-	-	-	-	-	-
**logFC: OA > YA**	**Sub-study 1**			**Sub-study 2**		
	*b**	*SE*	95% CI	*b**	*SE*	95% CI
(Intercept)	15.12	1.70	[11.54, 18.69]	32.34	2.10	[27.92, 36.76]
$logFC_{Fixels(OA>YA)}$	-6.37	20.93	[-50.53, 37.79]	2.89	30.83	[-61.89, 67.67]
$TIV_{Normalized}$	0.45	2.40	[-4.61, 5.52]	-1.78	3.06	[-8.21, 4.66]
Sex [female]	-2.05	1.78	[-5.80, 1.70]	-6.75	2.76	[-12.54, -0.96]
$Age_{mean\ centered}$	-	-	-	-1.18	0.53	[-2.30, -0.06]
**FDC: OA > YA**	**Sub-study 1**			**Sub-study 2**		
	*b**	*SE*	95% CI	*b**	*SE*	95% CI
(Intercept)	25.14	12.33	[-0.87, 51.14]	21.35	21.08	[-22.94, 65.63]
$FDC_{Fixels(OA>YA)}$	-13.95	16.63	[-49.04, 21.15]	15.41	29.18	[-45.90, 76.72]
$TIV_{Normalized}$	0.79	2.03	[-3.50, 5.07]	-1.82	2.75	[-7.61, 3.96]
Sex [female]	-2.02	1.74	[-5.68, 1.65]	-6.53	2.74	[-12.29, -0.77]
$Age_{mean\ centered}$	-	-	-	-1.17	0.49	[-2.19, -0.14]

Supplementary Table 17. Estimation of the model coefficients for the final models used to evaluate the regression of FBA-related metrics with BTT score in the older adults. FBA-metrics are calculated from the fixels that showed higher FDC values in older compared to younger adults. Abbreviations: b* = estimated regression coefficient, CI = confidence interval, FD = fiber density, FDC = fiber density and cross-section, logFC = logarithmic fiber cross-section, OA = older adults, SE = standard error, TIV = total intracranial volume, YA = younger adults.

5 References

Abraham, A., Pedregosa, F., Eickenberg, M., Gervais, P., Mueller, A., Kossaifi, J., et al. (2014). Machine learning for neuroimaging with scikit-learn. *Frontiers in Neuroinformatics* Volume 8 - 2014. doi: 10.3389/fninf.2014.00014.

Avants, B.B., Epstein, C.L., Grossman, M., and Gee, J.C. (2008). Symmetric diffeomorphic image registration with cross-correlation: Evaluating automated labeling of elderly and neurodegenerative brain. *Medical Image Analysis* 12(1)**,** 26-41. doi: https://doi.org/10.1016/j.media.2007.06.004.

Ciric, R., Thompson, W.H., Lorenz, R., Goncalves, M., MacNicol, E.E., Markiewicz, C.J., et al. (2022). TemplateFlow: FAIR-sharing of multi-scale, multi-species brain models. *Nat Methods* 19(12)**,** 1568-1571. doi: 10.1038/s41592-022-01681-2.

Esteban, O., Markiewicz, C.J., Blair, R.W., Moodie, C.A., Isik, A.I., Erramuzpe, A., et al. (2019). fMRIPrep: a robust preprocessing pipeline for functional MRI. *Nature Methods* 16(1)**,** 111-116. doi: 10.1038/s41592-018-0235-4.

Fonov, V.S., Evans, A.C., McKinstry, R.C., Almli, C.R., and Collins, D.L. (2009). Unbiased nonlinear average age-appropriate brain templates from birth to adulthood. *NeuroImage* 47**,** S102. doi: https://doi.org/10.1016/S1053-8119(09)70884-5.

Fox, J., and Weisberg, S. (2019). *An R Companion to Applied Regression.* Thousand Oaks CA: Sage.

Gorgolewski, K., Burns, C.D., Madison, C., Clark, D., Halchenko, Y.O., Waskom, M.L., et al. (2011). Nipype: A Flexible, Lightweight and Extensible Neuroimaging Data Processing Framework in Python. *Frontiers in Neuroinformatics* Volume 5 - 2011. doi: 10.3389/fninf.2011.00013.

Markiewicz, C.J., Esteban, O., Goncalves, M., Provins, C., Salo, T., Kent, J.D., et al. (2024). "fMRIPrep: a robust preprocessing pipeline for functional MRI". Zenodo).

Tournier, J.D., Smith, R., Raffelt, D., Tabbara, R., Dhollander, T., Pietsch, M., et al. (2019). MRtrix3: A fast, flexible and open software framework for medical image processing and visualisation. *Neuroimage* 202**,** 116137. doi: 10.1016/j.neuroimage.2019.116137.

Tustison, N.J., Avants, B.B., Cook, P.A., Zheng, Y., Egan, A., Yushkevich, P.A., et al. (2010). N4ITK: Improved N3 Bias Correction. *IEEE Transactions on Medical Imaging* 29(6)**,** 1310-1320. doi: 10.1109/TMI.2010.2046908.

Zhang, Y., Brady, M., and Smith, S. (2001). Segmentation of brain MR images through a hidden Markov random field model and the expectation-maximization algorithm. *IEEE Transactions on Medical Imaging* 20(1)**,** 45-57. doi: 10.1109/42.906424.
